# Preparation Method and Application of Porous Poly(lactic acid) Membranes: A Review

**DOI:** 10.3390/polym16131846

**Published:** 2024-06-28

**Authors:** Jinxing Zhao, Xianggui Liu, Xuelian Pu, Zetong Shen, Wenqiang Xu, Jian Yang

**Affiliations:** 1 Key Laboratory of Advanced Packaging Materials and Technology of Hunan Province, Hunan University of Technology, Zhuzhou 412007, China; x15630499835@163.com (J.Z.); xgliu507@outlook.com (X.L.); abdabd534@163.com (X.P.); ttsz966@sina.com (Z.S.); 13470776026@163.com (W.X.); 2 National & Local Joint Engineering Research Center for Advanced Packaging Material and Technology, Hunan University of Technology, Zhuzhou 412007, China

**Keywords:** PLA, porous membrane, tissue engineering, oil–water separation

## Abstract

Porous membrane technology has garnered significant attention in the fields of separation and biology due to its remarkable contributions to green chemistry and sustainable development. The porous membranes fabricated from polylactic acid (PLA) possess numerous advantages, including a low relative density, a high specific surface area, biodegradability, and excellent biocompatibility. As a result, they exhibit promising prospects for various applications, such as oil–water separation, tissue engineering, and drug release. This paper provides an overview of recent research advancements in the fabrication of PLA membranes using electrospinning, the breath-figure method, and the phase separation method. Firstly, the principles of each method are elucidated from the perspective of pore formation. The correlation between the relevant parameters and pore structure is discussed and summarized, subsequently followed by a comparative analysis of the advantages and limitations of each method. Subsequently, this article presents the diverse applications of porous PLA membranes in tissue engineering, oil–water separation, and other fields. The current challenges faced by these membranes, however, encompass inadequate mechanical strength, limited production efficiency, and the complexity of pore structure control. Suggestions for enhancement, as well as future prospects, are provided accordingly.

## 1. Introduction

In recent years, there has been rapid progress in the development of high-performance porous membrane materials, leading to an increased utilization of these membranes. As a result, there is a growing demand for enhanced membrane functionality, emphasizing the significance of creating porous membranes that demonstrate exceptional performance. When selecting porous membrane materials, a wide array of options is available. Presently, commonly employed separation membrane materials mainly consist of petroleum-derived polymers such as polyvinylidene fluoride (PVDF) [1,2], polypropylene (PP) [3,4], polysulfone (PSF) [5,6], and polyether sulfone (PES) [7,8]. However, these petroleum-based polymers pose challenges in terms of post-use degradation and have the potential to contribute to environmental pollution. PLA is a linear thermoplastic biodegradable polyester that can be directly synthesized through the polymerization reaction of lactic acid which can be derived from renewable plant resources [9]. Table 1 provides the advantages and limitations of PLA porous membranes.

**Figure 1:**
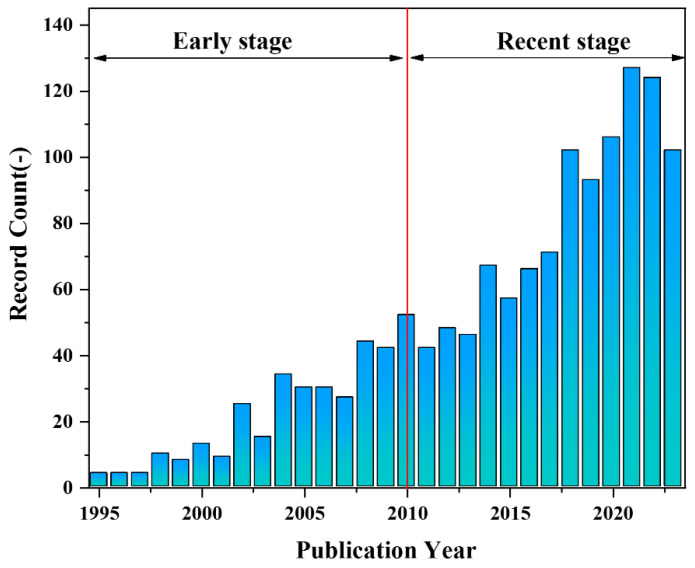
The count evolution of publications obtained from the “Web of Science” database using “PLA porous membrane Paper & Patent” as key title terms.

The PLA membrane can undergo complete degradation by microorganisms in the natural environment, making it a renewable and environmentally friendly material for various applications. Additionally, it exhibits excellent strength, processability and favorable biocompatibility, rendering it highly promising for tissue engineering [16,17,18], vascular transplantation [19,20,21], drug carrier [22,23,24], and other fields [15,25,26,27]. Zhong et al. [28] utilized PLA, nano-SiO_2_ and a mixture of good and poor solvents to fabricate superhydrophobic PLA film with nano/microstructured morphology using the non-solvent-induced phase separation (NIPS) method. The maximum water contact angle achieved was 164° ± 2.3°. The high hydrophobicity of the membrane holds significant potential in the realm of oil-water separation Liu et al. [29] immobilized a polydopamine (PDA) biological coating and silver nanoparticles (AgNPs) onto the membrane surface, followed by hydrophobic treatment with fluorinated mercaptan, resulting in the successful construction of a hierarchical rough structure of PLA. The water contact angle on the PLA membrane surface reached 158.6°, achieving a maximum permeate flux of 2664 L/(m^2^·h). Moreover, the separation efficiency for oil-water mixtures and water-in-oil emulsions exceeded 98.4%, demonstrating excellent stability and antibacterial properties. Hu et al. [30] utilized electrospinning technology to fabricate a composite nanofiber scaffold consisting of PLA, hydroxyapatite (HA), and polydopamine (PDA). Their findings revealed that the incorporation of HA-PDA particles significantly enhanced the hydrophilicity of the scaffold material. Moreover, compared to pure PLA nanofibers, the PLA/HA/PDA composite nanofiber scaffolds exhibited notably improved cell adhesion rate and survival rate. Consequently, these biocompatible (PLA)/HA/PDA composite nanofibers hold great potential as scaffolds for bone tissue engineering. Zhou et al. [31] employed electrospinning technology to fabricate a three-dimensional network structure of PLA/Polypyrrole (PPy) nanofiber scaffolds. In vitro experiments have demonstrated that the scaffold exhibits exceptional biocompatibility with human umbilical cord mesenchymal stem cells as well as Schwann cells, thereby enhancing their adhesion behavior. The PLA/graphene oxide (Go) drug-loaded nanofibers with different structures (single-axial and co-axial structure) were prepared by the electrospinning method, as demonstrated by Mao et al. [32]. A comparison of the drug release properties between PLA/Go nanofibers with different structures revealed that co-axial structure PLA/Go nanofibers exhibit enhanced stability and durability in drug release performance, effectively preventing sudden drug release. Consequently, these nanofibers hold significant potential for broad applications in the field of controlled drug release. Buscemi et al. [33] connected α,β-poly(N-2-hydroxyethyl)-D,L-aspartamide (PHEA) with PLA and blended it with polycaprolactone (PCL) to formulate an electrospinning solution. Subsequently, the electrospinning technology was employed to prepare a vascular stent exhibiting excellent mechanical strength and elasticity, along with favorable biocompatibility.single-axial and co-axial structure. Scientific research reports on the properties and potential applications of PLA porous membranes have consistently increased over the past three decades, as depicted in Figure 1.

The incorporation of biodegradable PLA as a substrate for crafting porous membrane materials not only overcomes challenges related to molding but also facilitates swift degradation by microorganisms into carbon dioxide and water upon disposal. This effectively addresses environmental pollution concerns, establishing it as a pivotal focus in research. The investigation and implementation of porous PLA membranes in tissue engineering and oil-water separation are poised to propel the progress of sustainable industry chains within the polymer materials domain. As a result, this confers notable research significance and practical value to the preparation of such membranes.

In this section, we propose the utilization of PLA, a biodegradable material, as one of the solutions in response to environmental pollution caused by petroleum-based polymers. A comprehensive analysis is provided on the origin, properties, and primary application domains of PLA materials

## 2. Preparation Method of PLA Porous Membranes

The current techniques employed for fabricating PLA porous membranes include electrospinning, breath figure technology, and phase separation technology. In this section, a succinct discussion is provided on the diverse techniques employed in the preparation of PLA porous membranes, placing particular emphasis on conducting a comparative analysis of their respective advantages and limitations.

### 2.1. Electrospinning

**Figure 2:**
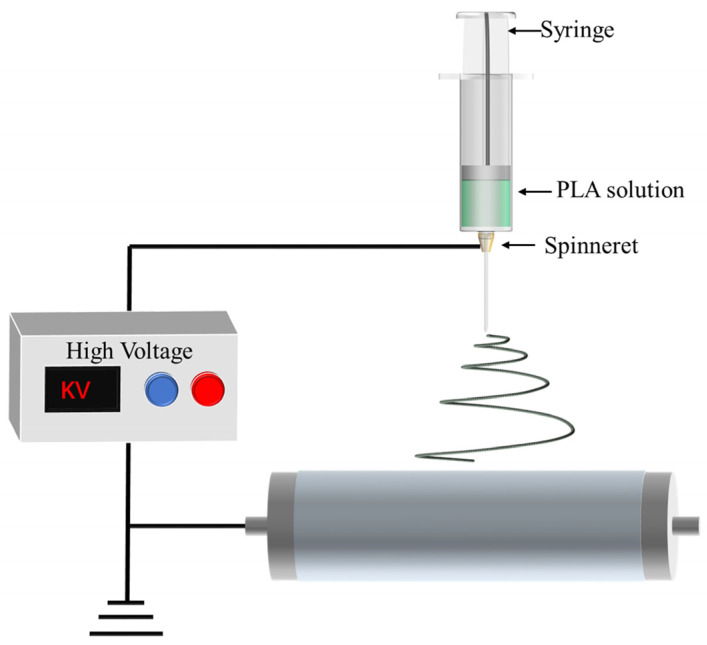
Process diagram of preparing a PLA porous membrane by electrospinning technology.

Electrospinning stands out as one of the most frequently employed techniques for producing PLA fiber membranes. The primary preparatory steps involve formulating a PLA spinning solution and subsequently injecting it into the spinning machine. The fundamental principle is to enhance the surface area of the spinning solution in order to induce an electric current through a high-voltage electric field. This leads to multiple stretching and splitting of the spinning solution during the injection process, as it shifts along a spiral path towards the receiving device for solidification into nanofibers. The overlapping of numerous fibers results in the formation of a porous film [34,35,36]. The schematic in Figure 2 illustrates the electrospinning process. By adjusting various electrospinning factors, such as voltage, spinneret diameter, working distance between the spinneret and receiving substrate, and other polymer parameters specific to PLA electrospinning, the manipulation of the structure and properties of PLA nanofibers becomes feasible [37,38]. Table 1 provides a summary of electrospinning parameters and pore sizes for different PLA based polymers.

In addition to the conventional nonwoven structure of PLA nanofibers, special functional PLA nanofibers can be prepared based on various polymer parameters and process conditions of PLA electrospinning. Examples include porous structure PLA nanofibers [39,40,41], and shell-core structure PLA nanofibers [42,43,44]. These unique structures possess characteristics that find application in diverse fields, garnering significant attention from researchers worldwide. This section introduces the porous structure and shell-core structure of PLA nanofibers.

#### 2.1.1. PLA Nanofibers with Porous Structure

The high porosity and specific surface area of PLA nanofibers confer novel properties upon nanofiber materials [39,45]. Consequently, the electrospinning method for producing PLA nanofibers with a porous structure has captured significant attention from researchers. The choice of solvent during the electrospinning process plays a pivotal role in achieving a porous structure, with highly volatile solvents in the spinning solution contributing to the formation of a porous surface on the fibers.

Yang et al. [41] employed chloroform as a solvent in the electrospinning process to fabricate PLA nanofibers, observing the development of porous structures on nanofibers with diameters ranging from 100 to 400 nm. These porous structures notably enhanced the hydrophobicity of the PLA nanofibers. Tian et al. [39] immersed PLA electrospun nanofibers in acetone at 4 °C for 20 h, resulting in a highly porous structure on the nanofiber surface and an increase in specific surface area from 18.4 m^2^/g before immersion to 137.7 m^2^/g. Additionally, solid-state phase separation can be employed to produce PLA nanofibers with a multi-pore structure. Bognitzki et al. [40] crafted composite nanofibers of PLA/polyvinylpyrrolidone (PVP) using the electrospinning technique, followed by the selective removal of PVP in water, which process resulted in the formation of porous PLA nanofibers suitable for applications as adsorption materials.

#### 2.1.2. PLA Nanofibers with Shell-Core Structure

The core/shell structure fiber is a composite fiber distinguished by its double or even multi-layered architecture, where the core and shell comprise components with distinct structures and physical properties. The fabrication process of PLA nanofibers with a shell-core structure not only incorporates the advantages of large specific surface area, high porosity, and small diameter typical of nanofiber materials but also facilitates the production of multifunctional nanofiber materials through the inclusion of an additional component [43,44].

**Figure 3:**
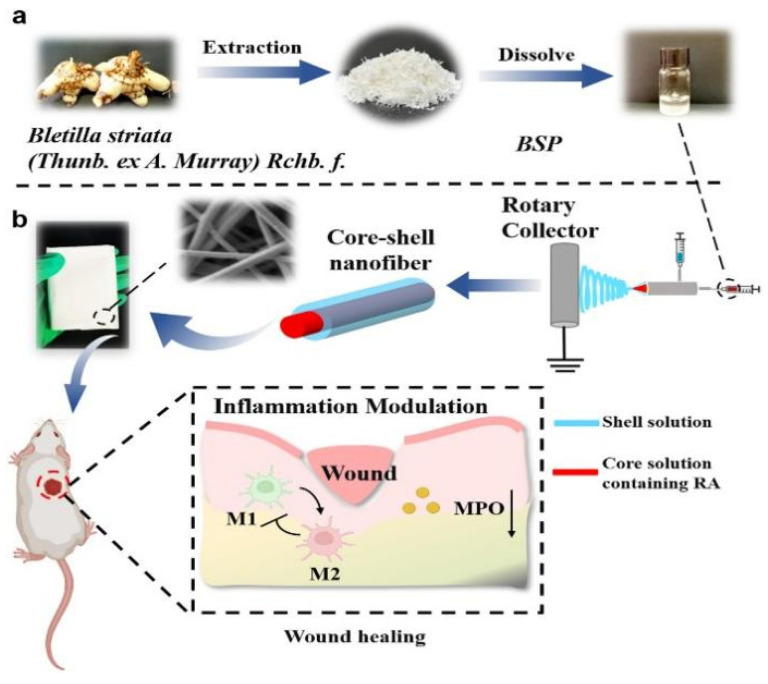
RA-BSP-PVA-PLA coaxial nano-fiber design process and schematic diagram to promote wound healing [42]. (**a**) Preparation and application of RA-BSP-PVA@PLA nanofibers, extraction and preparation of BSP. (**b**) Schematic diagram of preparation of coaxial nanofibers and their promotion of wound healing.

Zhong et al. [42] produced a core-shell nanofiber material using Bletilla *striata polysaccharide* (BSP)-Polyvinyl alcohol (PVA)-PLA, as depicted in Figure 3 to illustrate the design strategy of the nanofibers.The formation of the core-shell structure was successfully confirmed through Confocal laser scanning microscopy (CLSM) and Fourier transform infrared spectroscopy (FTIR). Rosmarinic acid (RA)-BSP-PVA@PLA exhibits excellent biocompatibility. Moreover, the incorporation of BSP and RA significantly enhances the mechanical properties of coaxial fibers, possibly due to an increased number of intermolecular hydrogen bonds. Chen et al. [43] created core-shell porous nanofibers loaded with drugs using coaxial electrospinning and non-solvent-induced phase separation, employing polycaprolactone/PLA as the materials. In comparison to porous nanofibers prepared by single-axis electrospinning, these coaxial porous nanofibers exhibit improved control over drug release rates while promoting it. Tian et al. [44] utilizing nerve growth factor (NGF) and Silk Fibroin (SF) as the core layer and PLA as the shell layer, through coaxial electrospinning, prepared composite nanofibers with a shell structure for use as cell scaffold materials. The results demonstrate that the scaffold not only slowly releases NGF and prolongs NGF viability, but also promotes adhesion and proliferation of rat cells, crucial for advancements in the field of biological medicine. Table 2 provides the effects of different polymer types, solvent types, voltages, and tip-Collector Distance on the pore structure.

Electrospinning can be utilized for the large-scale production of porous PLA membranes. However, it exhibits low production efficiency, a wide distribution of pore sizes, relatively high production costs, and suboptimal mechanical properties.

### 2.2. Breath-Figure Method

**Figure 4:**
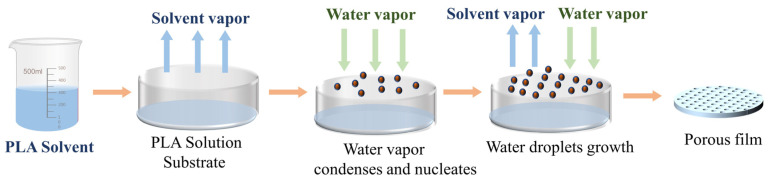
Process diagram of preparing a PLA porous membrane by BF method.

The phenomenon of Breath-Figure (BF) refers to the formation of water mist on solid or liquid surfaces, which was initially discovered and reported by Francois as the self-assembly of fine-scale polymer honeycomb structures facilitated by water [57]. The low surface temperature of a solid or liquid induces the release of heat from water vapor in the air, leading to its condensation on the surface. Due to the growth rate and mutual forces, organized arrangements of water droplets develop, acting as templates for surrounding solid material to solidify into regular patterns. Figure 4 illustrates the fabrication process of PLA porous membranes utilizing this technique.

The BF method provides the advantage of not requiring additional template removal while allowing simultaneous morphological construction and functional modification in a single step. This imparts convenience and efficiency compared to other methods for preparing porous films. The preparation conditions exhibit a linear relationship with the regular morphology and size, enabling easy control of pore sizes [58,59,60]. Consequently, an increasing number of researchers are employing the BF self-assembly technique to fabricate mesoporous films, finding extensive applications in fields such as bioengineering, optical devices, and sensing [61,62,63,64]. Preuksarattanawut et al. [65] successfully prepared a cellular-patterned porous biodegradable PLA membrane using the BF method. Their investigation considered the impact of initial polymer concentration in the solution, solvent type, and relative humidity of the airtight chamber on the pore size of the membranes. The findings suggest that a highly ordered porous membrane with an average pore size of 23.29 ± 4.55 μm can be achieved by employing a high PLA concentration (10% wt PLA) in dichloromethane at a relative humidity range of 80–85%.

However, directly preparing ordered porous films of PLA only with PLA by BF is challenging. In general, it is necessary to chemically or physically modify the PLA, for example, through chemical copolymerization. Copolymerization proves to be the simplest and most effective method for synthesizing new PLA composite materials. Li et al. [58] successfully synthesized structurally controllable polymethylene (PM)/poly (D, L-lactide) diblock copolymers (PM-*b*-PLA). These block copolymers, synthesized by combining the living polymerization of ylides with the ring opening polymerization of D, L-lactide, can be prepared into ordered porous films through the BF method. Although the PLA homopolymer film prepared by the BF method did not exhibit a regular porous structure, the film prepared by PM-*b*-PLA_2_ displayed a higher ordered honeycomb-like porous structure compared to the PLA homopolymer film, with a pore size of approximately 4.8 µm. The presence of PM segments reduces the hydrophilicity of PM-*b*-PLA_2_ and its solubility in dichloromethane, facilitating the formation of ordered porous structures. Bertrand et al. [59] successfully synthesized PLA-*b*-PS diblock copolymers via controlled polymerization, creating a block copolymer (BCP) capable of forming cylindrical structures. By combining the rapid solvent evaporation BF method with additional nanoscale self-assembly of PLA-*b*-PS diblock copolymers, they prepared a hierarchically porous polymer film with a highly regular honeycomb-like microporous structure.

**Figure 5:**
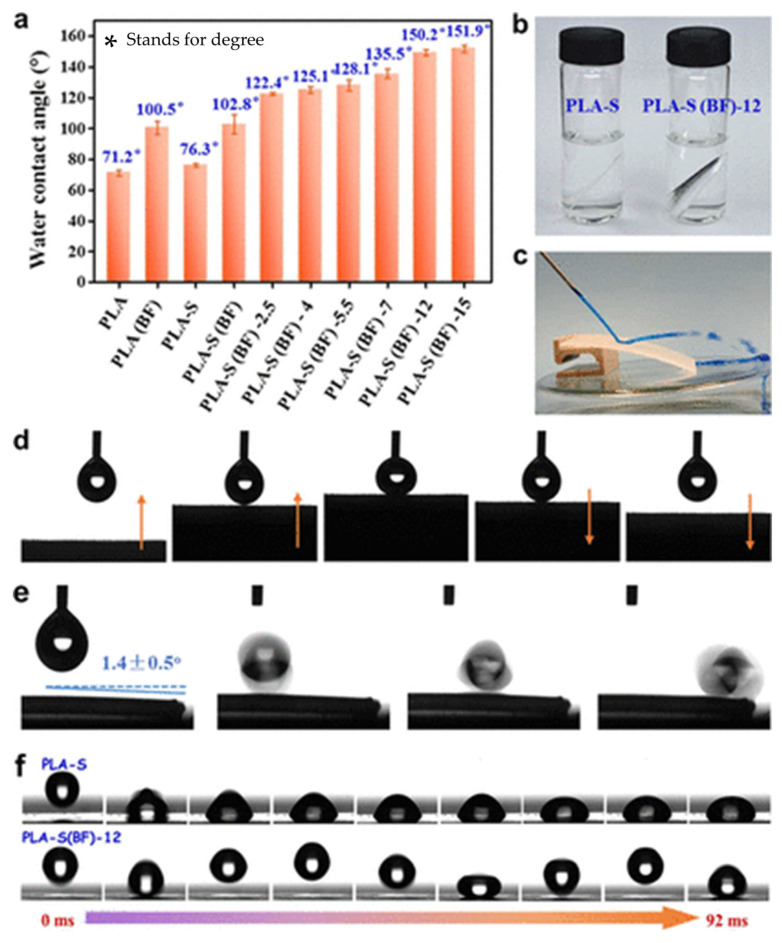
(**a**) WCA of the oriented PLA films before and after surface modification. (**b**) Digital photograph of PLA-S and PLA-S(BF)-12 samples immersed in water. (**c**) Water jet bouncing off from the PLA-S(BF)-12 film surface. (**d**) Low-adhesive surface of the PLA-S(BF)-12 film. (**e**) Rolling of the water droplet on the PLA-S(BF)-12 film with a small tilt angle. (**f**) Droplet pinning and rebounding behavior when released from a height of 0.5 cm toward PLA-S and PLA-S(BF)-12 films [60]. (PLA-S stands for thin film prepared by method casting-thermal stretching; PLA-S(BF) stands for thin films that have been surface modified by the BF method and FAS-SiO_2_; 12 stands for 12 mg/mL of FAS-SiO_2_ suspensions; FAS-SiO_2_, fluorinated silica nanoparticles).

Blending proves to be an effective method for modifying PLA and preparing porous membranes. Chen et al. [60] fabricated relatively ordered microporous structures using the BF method and introduced hydrophobic silica nanoparticles to construct micro/nanostructured surfaces with low surface energy. As depicted in Figure 5, a superhydrophobic PLA film exhibiting high water contact angle (WCA) of 150.2° and low sliding angle of 1.4° could be achieved when the nanoparticle content reached 12 mg/mL. Moreover, owing to the exceptional crystallinity and stability of silica particles, the PLA film exhibited remarkable self-cleaning properties as well as resistance against heat, acid, and water. This study offers a straightforward approach with industrial potential for manufacturing PLA materials with unique wettability properties and exceptional mechanical characteristics, suitable for applications in self-cleaning and moisture-proof packaging. Guo et al. [66] fabricated PLLA (poly(L-lactide))/Carbon Quantum Dots (CQD) porous membranes using a CQD-assisted static breathing method, wherein agricultural waste was transformed into high-value CQDs. The excellent dispersion of CQDs in water significantly enhanced the stability of water droplets on the surface of the PLA solution, facilitating the creation of a porous structure. Moreover, CQD demonstrated good degradability towards PLA. This study offers cost-effectiveness, ease of operation, and high versatility, effectively addressing limitations such as high humidity, air flow, and amphiphilic surfactants. Table 3 summarizes the effects of different polymer types, relative humidity levels, and solvent types on pore structure.

### 2.3. Phase Inversion Method (PI)

The phase inversion (PI) method, initially proposed by Leob et al. in 1964, currently stands as the most widely employed technique for fabricating polymer porous membranes [73]. Owing to its versatility and scalability, the phase inversion method has emerged as the predominant approach due to its simplicity and adaptable production scales. Based on the disparities in the thermodynamic conditions of polymer solutions, and there are four primary methods for phase inversion: thermally induced phase separation (TIPS), non-solvent-induced phase separation (NIPS), vapor-induced phase separation, and solvent evaporation phase inversion method [74,75,76]. Among these, the most frequently employed phase inversion techniques are TIPS and NIPS. Vapor-induced phase separation method is commonly utilized for preparing honeycomb block copolymer films, requiring specific polymer-solvent combinations. In contrast, the solvent evaporation phase inversion method is often chosen for producing polymer due to its prolonged film-forming duration and comparatively compact film structure. TIPS and NIPS are described in detail below.

#### 2.3.1. Thermally Induced Phase Separation

**Figure 6:**
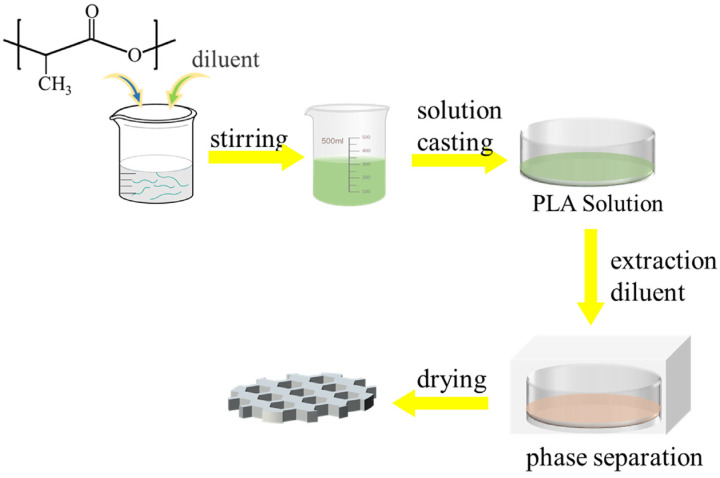
Schematic diagram of the film formation process of porous PLA prepared by TIPS method.

The TIPS process involves the creation of a homogeneous and stable casting film solution at high temperatures, where a polymer and a diluent with a high boiling point are combined. The homogeneous casting film liquid undergoes phase separation and polymer solidification, leading to the extraction of the diluent and the formation of a porous film [77,78,79,80,81,82,83]. Figure 6 provides a schematic diagram of the TIPS process.

**Figure 7:**
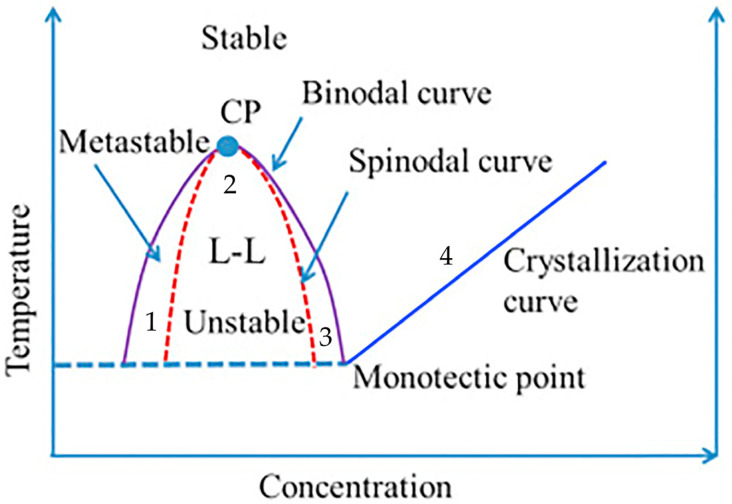
Thermally induced phase separation binary phase diagram [86]. The abbreviation CP refers to the critical temperature.

The phase diagram serves as a fundamental tool for investigating the thermodynamic state of polymer solutions, enabling us to determine the suitability of a homogeneous polymer solution in a solvent for film formation [84]. A comprehensive phase diagram illustrating the TIPS method is presented in Figure 7. 

The liquid-liquid separation region and solid-liquid separation region are demarcated by dynamic crystallization boundaries. The point of intersection between the binodal curve and the dynamic crystallization boundary is known as the monotectic point, which signifies the critical polymer concentration that distinguishes liquid-liquid phase separation from solid-liquid phase separation in the system. The polymer concentration for cooling paths 1, 2, and 3 is lower than the concentration at the monotectic point, resulting in liquid-liquid phase separation and leading to the formation of a bicontinuous film structure. The concentration of the polymer in path 4, however, surpasses the critical monotectic point concentration, leading to a phase separation between solid and liquid phases. As a result, a distinct spherical accumulation structure becomes visibly apparent in the solidified film. The absence of liquid-liquid phase separation is indicated [85]. Generally, as the polymer content gradually increases in the casting liquid system, the resulting membrane structure becomes more densely packed with reduced porosity and pore size [86].

**Figure 8:**
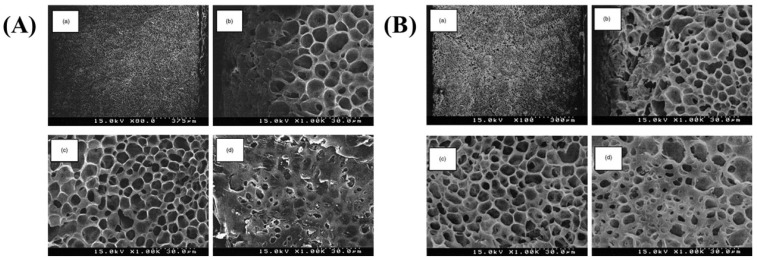
Morphology of different cooling rates (**A**). Water from 80 to 0 °C. (**B**) Water from 50 to 0 °C. (**a**) Overview; (**b**) near the top side; (**c**) near the center; (**d**) the top surface [88].

The porosity morphology of the membrane in the TIPS method for porous membrane fabrication is influenced by the thermodynamic state of the solution. Modifying freezing temperature, solution composition, and concentration can effectively alter the porous morphology [87]. Tanaka et al. [88] initially fabricated PLLA microfiltration membranes using a thermally induced phase separation technique in 2004. They conducted an investigation on the impact of various cooling rates on the morphology of porous membranes, as depicted in Figure 8, and observed that higher cooling rates tend to generate anisotropic pore structures, whereas lower cooling rates lead to pore structures closer to isotropic.The presence of anisotropic pore structures can significantly enhance the permeation flux of microfiltration membranes. Fariba et al. [89] conducted a study on porous PLLA membranes using liquid-liquid and TIPS procedures to investigate the impact of quenching time, temperature, and film thickness on morphology, water absorption and mechanical properties. The findings suggest that an elevated freezing temperature can increase membrane porosity due to a reduced cooling rate and solvent nucleation rate within the specified thickness. Hou et al. [14] successfully fabricated a novel porous PLA film through blade coating and thermally induced phase separation. The leaf vein-like-oriented pores were obtained by enhancing the crystallization capacity of the stereoscopic composite crystals. The films exhibited an elongation at break of 45.5% and a porosity of 90.1%. Furthermore, the enhanced crystallinity significantly enhances the thermal resistance of the film Table 4 summarizes the preparation parameters of PLA membranes fabricated via the TIPS method.

#### 2.3.2. Non-Solvent-Induced Phase Separation

**Figure 9:**
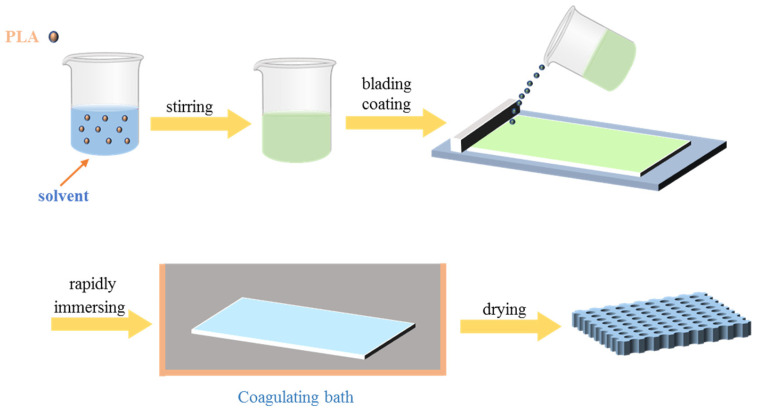
Schematic diagram of the film formation process of porous PLA prepared by NIPS method.

The NIPS technique is widely employed in the field of phase inversion membrane technology. NIPS typically occurs at ambient temperature through the immersion of a homogeneous casting polymer solution into a non-solvent. The mass transfer between the solvent and non-solvent at the phase interface of the casting solution results in phase separation, polymer solidification, and the formation of a porous membrane. Figure 9 provides a schematic illustration of the non-solvent phase separation process.

**Figure 10:**
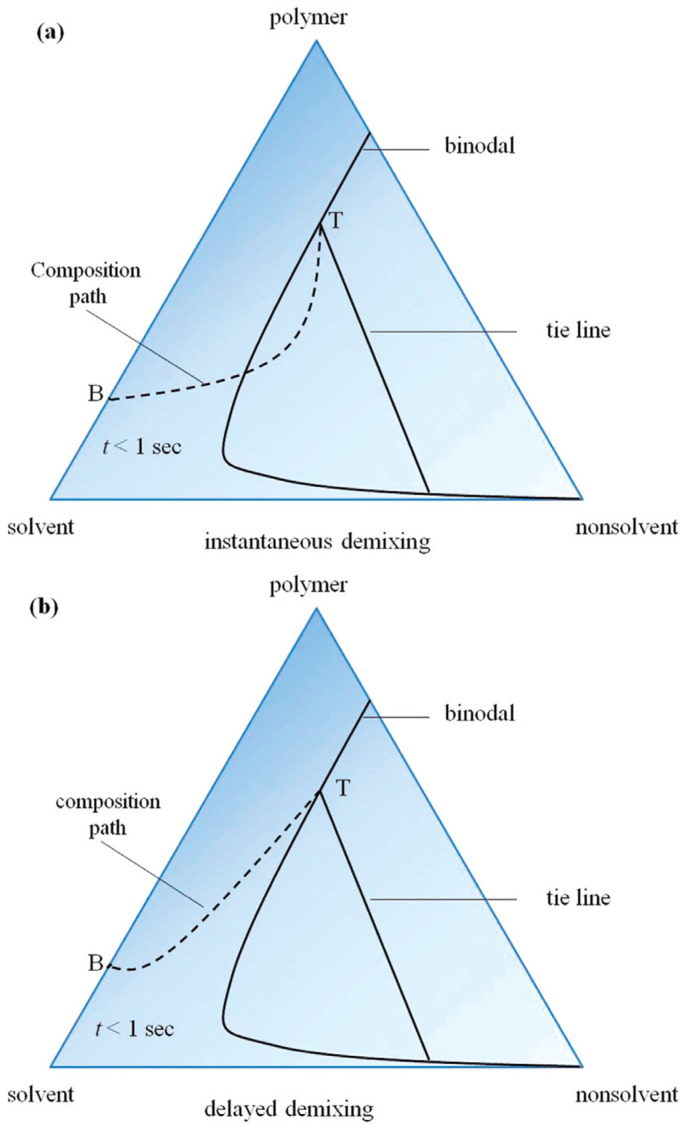
Non-solvent phase separation process: (**a**) path 1, (**b**) path 2 [102].

The investigation of the thermodynamic phase diagram of the film-forming system facilitates the analysis and prediction of its film formation behavior [101]. The film formation process of the non-solvent phase separation method can be elucidated by referring to the ternary phase diagram illustrated in Figure 10. By manipulating the thermodynamic state and exerting control over phase transformation, precise manipulation of the film formation structure can be achieved. The immersion process of the primary film of polymer solution composed of B into a non-solvent bath may involve two distinct paths, namely path 1 and path 2. The path 1 involves passing through the two-node line to access the two-phase region, resulting in liquid-liquid phase separation and ultimately obtaining a membrane with a porous surface. The polymer solution undergoes a glass transition and directly enters the glass state for path 2, without traversing the two-node line. The film surface undergoes a transformation into a uniformly dense structure in this state.

The microstructure of PLA film is determined by the competitive outcome of phase separation, as dictated by the NIPS film forming mechanism, which is significantly influenced by the solvent/non-solvent system. Xing et al. [75] prepared PLA membranes by utilizing a coagulation bath consisting of a mixture of ethanol and water in varying proportions. They subsequently investigated the influence of the components within the coagulation bath on membrane structure, crystallinity, and porosity. The spongy structure was significantly influenced by the moisture content in the coagulation bath, with an observed disruption of the homogeneous spongy structure as the moisture content increased. However, accurately controlling the morphology of the PLA membrane proved challenging due to the difficulty in controlling the exchange rate between the solvent and non-solvent during the phase transition. To address this limitation, Hu et al. [103] proposed a novel approach to regulate the rate of solvent-to-non-solvent exchange by transforming non-solvent water from a continuous liquid phase into liquid microdroplets using an ultrasonic generator. In their study, the involvement of non-solvent states in the induced phase transition occurred in the form of microspheres, attenuating the exchange rate between solvent and non-solvent during the phase transition process and prolonging the curing time of the film. Moriya et al. [104] investigated the impact of different solvent types and PEG additives on film properties, finding that utilizing dimethyl sulfone as a solvent maintained the low viscosity of the casting film solution while resulting in a prepared film with excellent water permeability and ultrafiltration separation performance. The resulting membrane exhibited a membrane flux of 882 L/(m^2^·h·atm) and retained 80% of the bovine serum albumin (BSA) when employing 10% PEG. Table 5 summarizes the solvent and non-solvent preparation of PLA membranes by NIPS method and adds the necessary additional explanations in the comment column.

**Figure 11:**
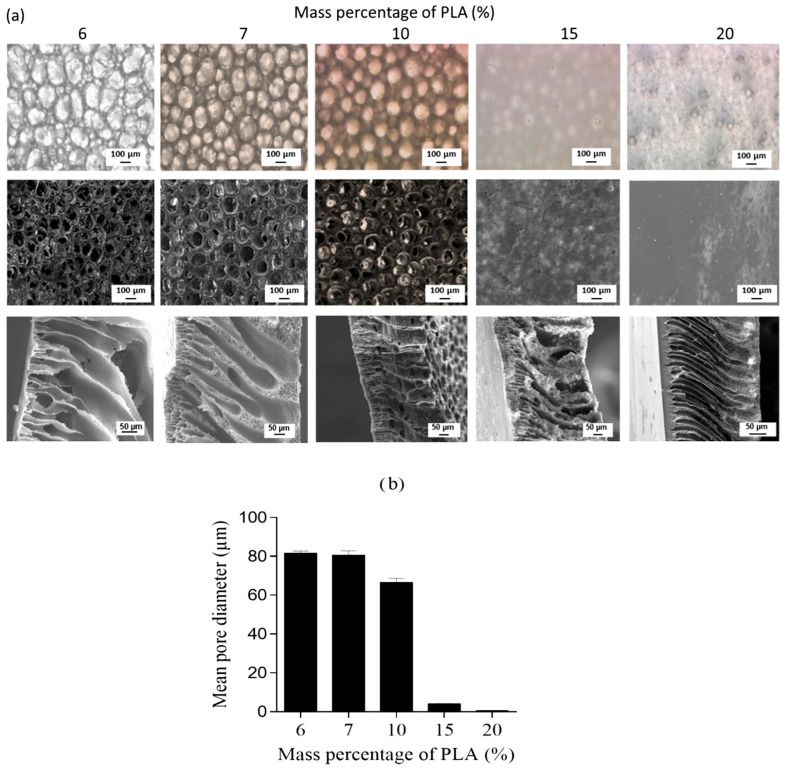
(**a**) Optical microscopy (**top**) and SEM (**middle**) views of the porous bottom surface and SEM views of the cross section (**bottom**) of PLA membranes. (**b**) Mean diameter (μm) of macropores open on the bottom surface of the membrane [105].

The concentration of the polymer in the casting solution will impact the morphology of the final film, as it is responsible for forming the membrane matrix [102]. The membrane porosity tends to decrease as the polymer concentration increases [105]. Additionally, when the polymer concentration is low, the casting liquid exhibits a reduced viscosity and enhanced fluidity. Consequently, the resulting film demonstrates excellent surface flatness and uniform thickness; however, it also possesses elevated porosity during film formation and compromised mechanical strength. The PLA solution with a mass percentage of 6%~20% (*w*/*w*) was prepared by Elas et al. [105] using the NIPS method. The impact of polymer concentration on the porous structure of the membrane was observed. As depicted in Figure 11, an increase in the mass percentage of PLA in the polymer solution resulted in a reduction in the number and average diameter of pores on the bottom surface, as well as a decrease in membrane porosity. The average pore size exhibits a significant decrease, particularly when the concentration of PLA solution exceeds 10% (*w*/*w*).

**Table 5:** Preparation parameters of PLA membranes fabricated via NIPS method.

	Species	Comments
Solvent	DCM [106,107,108,109]	Solvent should be compatible with coagulation media.
	NMP [11,13,110],	
	DMAc [111,112],	
	Acetic acid [113],	
	DMF [105,114,115,116],	
	DMSO [117]	
	1,4-dioxane [75,103]	
Non-solvent	Ethanol [109],	Due to cost considerations, non-solvents of PLA are often water.
	Hexane [107],	
	Water [11,13,106,110,112,113,114,115,116,117,118],	
	NMP [108]	

In summary, membranes can be prepared through methods such as electrospinning, breath figure method, thermally induced phase separation, and nonsolvent-induced phase separation. A comparison of these four methods is summarized in Table 6.

## 3. Applications 

**Figure 12:**
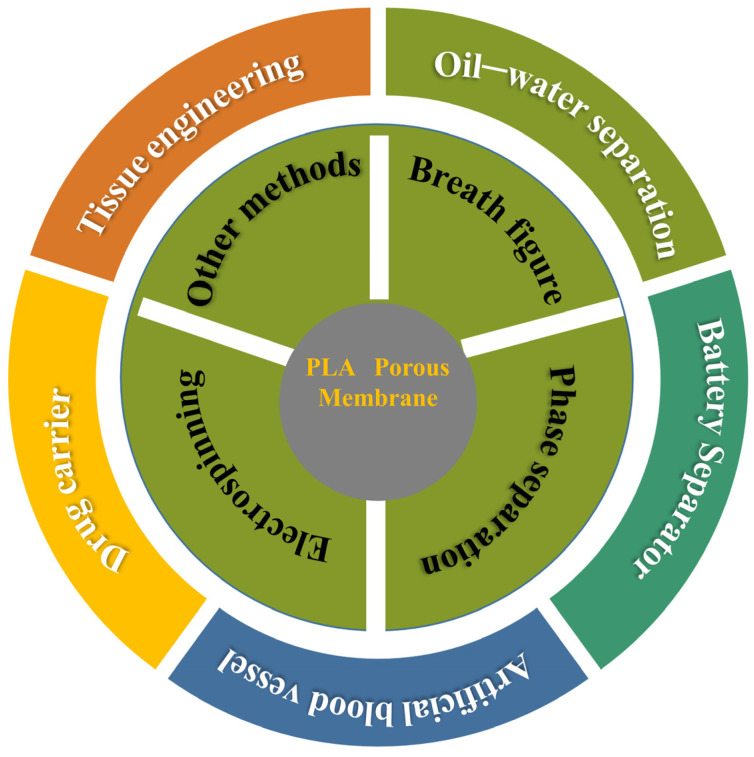
The universal application of PLA porous membranes prepared by different methods.

The excellent degradation properties and biocompatibility of PLA porous membranes have led to their wide applications in various fields, such as oil-water separation, tissue engineering, and drug delivery [120,121]. The following section primarily discusses the application of PLA porous membranes in the fields of tissue engineering and oil-water separation. The application scope of PLA porous membrane is depicted comprehensively in Figure 12.

### 3.1. Tissue Engineering

PLA possesses outstanding biocompatibility and biodegradability, and its degradation products are not significantly toxic to cells. It is recognized as a Food and Drug Administration (FDA) certified biomaterial for tissue engineering applications. PLA and its composite materials are currently widely utilized as biomaterials in tissue engineering. The interconnected network pore structure of PLA porous membranes demonstrates superior material transfer efficiency, providing ample internal space to support cell division and growth. Additionally, it promotes the flow and transport of nutrients and oxygen gas, along with the efficient removal of superfluous waste [122,123]. Pinto et al. [124] developed a composite membrane comprising PLA and graphene-based materials, specifically graphene oxide (GO) and graphene nanoplatelets (GNP), for tissue engineering applications. The incorporation of GO onto the membrane surface generated an appropriate surface topography and enhanced hydrophilicity, promoting cell adhesion and proliferation. The inclusion of GNP facilitated accelerated tissue regeneration while concurrently reducing thrombosis occurrence and postoperative complications. Li et al. [125] utilized the -COOH moiety of colorless 3-aminopropyltriethoxysilane (APTES) to react with the -NH_2_ group of heparin, immobilizing it onto the surface of PLA membrane through multiple self-condensation processes, including surface adhesion, polycondensation, and multilayer interaction. This approach effectively facilitates the surface heparinization of PLA porous membrane, enhancing its blood compatibility. Promnil et al. [126] successfully fabricated PLA/Silk Fibroin (SF) nanofiber scaffolds via the electrospinning technique. Their research findings indicate that viscosity plays a pivotal role in determining the ability, morphology, and size of fibers. The average fiber diameter increases proportionally with the increased viscosity of the solution. Three essential parameters influencing solution viscosity include concentration, structure, and molecular weight of PLA. Wang et al. [127] developed and fabricated a novel biocompatible PLA scaffold with a directional porous structure for bone tissue engineering using an ice template and the phase inversion technique. They discovered that the directional scaffold containing 8% of the material exhibited exceptional mechanical properties while maintaining a longitudinally connected porous structure with porosity ranging from 82% to 98%. This enhanced architecture facilitates efficient nutrient and cell penetration. Table 7 provides a summary of the applications of different PLA porous membranes in tissue engineering.

### 3.2. Oil–Water Separation

The severity of water pollution has been progressively escalating in recent years as a result of recurrent oil spills and the substantial release of industrial oily wastewater, thereby placing a considerable strain on the environment [135]. Conventional treatment methods include filtration, direct combustion, centrifugation, and electrochemical techniques [136,137,138,139]. The filtration method has gained widespread popularity due to its straightforward operation, exceptional efficiency, and cost-effectiveness. However, the majority of current membrane materials are non-degradable, posing a significant risk of secondary pollution. The incorporation of degradable polymers can effectively address this issue, rendering it imperative to opt for such materials in the fabrication of environmentally friendly oil-water separation materials.

PLA membranes exhibit high porosity and inherent hydrophobic properties, rendering them suitable for multiple recycling cycles following appropriate treatment. Consequently, the initial PLA oil-water separation material possesses superhydrophobic, superlipophilic, and biodegradable characteristics, making it an environmentally friendly separation material with significant developmental potential that can be fabricated through phase separation, electrospinning, and other techniques. Zhou et al. [140] successfully fabricated a hierarchical rough structure by depositing a titanium dioxide (TiO_2_) layer and methyltrichlorosilane (MTS) onto PLA nanofibers. The resulting film exhibited stable superhydrophobicity with a water contact angle of 157.4° ± 0.9° and demonstrated excellent resistance to water adhesion across different pH values. Moreover, the film displayed remarkable oil-water separation efficiency, achieving over 95% separation for various oils, making it highly suitable for efficient oil-water separation. Mo et al. [25] fabricated a hydrophobic PLA/carbon nanotubes (PLA/CNTs) composite fiber membrane via electrospinning, wherein the introduction and crystallization modification of carbon nanotubes resulted in a low polarity, rough, and porous surface that enhanced its hydrophobicity and oil-water selectivity. The prepared fiber film exhibited an oil absorption capacity of 114.01 g/g, a contact angle of water in air at 134.2°, and a contact angle of water under oil at 157.3°, indicating high separation efficiency for oil-water mixtures and making it a promising candidate for applications in oil-water separation. Xin et al. [141] utilized a simple slow solvent-evaporation induced precipitation method to fabricate porous PLA material with adjustable microstructure, and discovered that compared to solvent-casting bulk PLLA film, the porous PLA material exhibited higher contact angle (121°) and porosity (48.4%). Consequently, PLA porous material holds promising prospects in the field of oil-water separation. The oil-water separation performance of PLA-based materials is summarized in Table 8.

### 3.3. Other Applications

#### 3.3.1. Porous Polymer Electrolytes

Advancements in portable electronic devices, wearable technology, electric vehicles, and energy storage systems are driving increasing demands for battery performance [27,152]. Lithium-ion batteries offer numerous advantages, such as a high operating voltage, an extended cycle life, minimal self-discharge, absence of memory effect, and more [152,153]. The separator plays a crucial role in lithium-ion batteries, serving two primary functions. Firstly, it acts as a physical barrier between the positive and negative electrodes, effectively prevent short circuits and ensure the battery’s safe operation. Secondly, it facilitates efficient ion transfer during both charging and discharging processes, guaranteeing the battery’s normal functionality. Therefore, extensive research on separator-related matters is imperative for advancing towards the next generation of high-performance battery systems since the separator significantly influences both safety and ion transfer in batteries.

**Figure 13:**
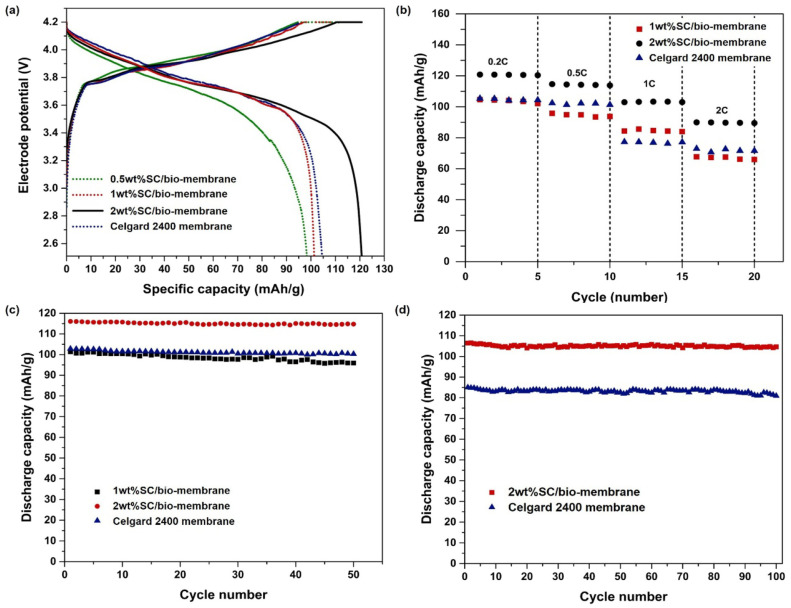
(**a**) Initial-cycle charge–discharge curves, (**b**) C-rate performance of cells with various membranes, and discharged cell cycling performance at (**c**) the 0.5C rate and (**d**) 1C rate [15].

To mitigate the environmental impact of polymers used in energy storage systems, Barbosa et al. [27] proposed a lithium-ion battery membrane based on poly-L-lactic acid (PLLA). They utilized thermally-induced phase separation to fabricate PLLA membranes with varying polymer mass concentrations (8% to 12%). The microstructure and morphology of the membrane were found to be influenced by the polymer concentration, leading to the emergence of macroscopic cavities as the concentration increased. Moreover, changes in polymer concentration minimally affected the thermal properties and dimensions of the membrane. In terms of battery performance, the PLLA membrane with an 8% concentration exhibited a discharge capacity of 23 mAh/g at a 1C rate, while the PLLA membranes with concentrations of 10% and 12% demonstrated capacities of 93 mAh/g and 82 mAh/g, respectively. This study compared the efficiency of this PLLA separator with other natural polymers and commercial separators, highlighting its suitability for battery applications. MCC with high porosity and hydrophilicity was incorporated into the PLA/PBS biocomposite membrane through non-solvent phase separation, as reported by Thiangtham et al. The findings demonstrated that the composite membrane containing 5% MCC exhibited superior electrolyte infiltration and absorption rates, along with enhanced ionic conductivity (2.06 mS/cm) and reduced interface impedance (1115 Ω) [116]. Additionally, Thiangtham et al. [15] utilized phase inversion technique to blend sulfonated cellulose (SC) with PLA/PBS composites for the fabrication of lithium-ion battery separators with varying SC content. The incorporation of SC enhances the thermal stability and wettability of the biomembrane. Figure 13 illustrates a comparison in electrochemical performance between a battery assembled with a PLA/PBS/SC separator and one using Celgard 2400 separator. The biomembrane separators exhibit superior reversible capacity, enhanced rate performance, and excellent cycle stability at a low cost while also being environmentally friendly.

#### 3.3.2. Drug Delivery 

The future prospects of polymeric materials in drug delivery technologies appear to be more promising compared to other materials such as nanocrystals and metal-organic frameworks [22]. The compatibility of PLA with human tissues is exceptional, and it undergoes gradual degradation in the body, ultimately converting into carbon dioxide and water. Moreover, the intermediate product of PLA is lactic acid (LA), a normal sugar metabolite that does not accumulate in vital human organs [13].

The influence of solvent ratio (DCM/acetone) on the pore structure and antibacterial activity of fiber membranes was investigated by Seo et al. [24] using the liquid-liquid phase separation technique. The study revealed that increasing the DCM amount from 50% to 70% in the polymer-solvent system resulted in an increase in average fiber diameter from 1.87 μm to 2.43 μm, leading to the formation of a more interconnected structure with numerous pores. This interconnected porous structure facilitates maximum gentamicin adsorption, enabling slow and sustained drug release, thereby optimizing patient treatment and providing long-term utility. In order to achieve effective control over drug release, Ju et al. [37] employed electrospinning and phase separation techniques for the preparation of porous PLA fiber films. The release behavior of ketoprofen was investigated at different temperatures, revealing a relatively high release rate at 37 °C.

#### 3.3.3. Artificial Blood Vessel

The recognition of cardiovascular disease as one of the leading causes of rising global mortality highlights its significant impact on individual health and lives, posing a formidable threat [154]. A comprehensive treatment plan for cardiovascular disease includes adherence to a nutritious diet, regular physical activity, and appropriate medication. In addition, certain patients may find relief through various chemical or physical interventions such as anticoagulant therapy for blood clot management or the surgical placement of a stent [155]. PLA has achieved significant advancements in the field of artificial blood vessels in recent years. 

Pyzik et al. [156] investigated the use of heparin-modified bilayer PCL and PLA-based scaffolds for minor vessel tissue engineering. The findings suggest that the combination of thermally induced phase separation and electrospinning yields asymmetric scaffolds with enhanced mechanical properties. The discharge of heparin from the stent was confirmed by both direct and indirect tests. The endothelial cell culture test showed that the human aortic endothelial cell lines (HAEC) activity of the heparin-modified scaffolds was higher with longer culture time. Therefore, the design and composition of the proposed stent have prospective applications in small vessel tissue engineering. The surface of PLA was modified using radio frequency plasma surface activation with perfluorinated compounds by Khalifehzadeh et al. [157], resulting in a reduction in thrombus formation and platelet reactivity, thereby enhancing its blood compatibility. The electrospinning technique was employed by LeyvaVerduzco et al. [158] to fabricate a tubular membrane composed of a low cytotoxic blend of PLA and collagen. Additionally, the cell viability assessment conducted on Human Umbilical Vein Endothelial Cells (HUVECs) demonstrated that the blended samples exhibited enhanced cellular activity with minimal toxicity.

The PLA porous membrane has gained extensive utilization due to its biodegradability, non-toxic nature, biocompatibility, and renewability. This section primarily focuses on its key applications in tissue engineering and oil-water separation, followed by a comprehensive exploration of its diverse applications in other domains.

## 4. Summary and Prospect 

The latest advancements and applications of the main methods for fabricating PLA porous membranes are comprehensively reviewed in this paper. Firstly, the performance of PLA as a porous membrane material is meticulously summarized from the perspective of porous polymer membranes, highlighting its distinct advantages over petroleum-based polymers, such as exceptional biocompatibility and biodegradability. Subsequently, we discussed the principles behind different methods and summarized the performance of PLA porous membranes and their latest research progress under various preparation methods. Additionally, through a comparative analysis of three methods, electrospinning and phase separation were found to be the most commonly used techniques for fabricating PLA porous membranes. Finally, based on the unique properties of PLA porous membranes, we primarily elucidated their diverse applications in tissue engineering, oil-water separation.

Although some progress has been made in the development of PLA porous membranes in recent years, numerous challenges still persist. Firstly, the mechanical properties of PLA porous membranes are generally subpar due to their high pore structure; thus, enhancing their mechanical properties holds significant importance. Modification technology is anticipated to enhance the mechanical properties of porous membranes. PLA modification can be achieved through three approaches: copolymerization modification, where other flexible molecular chain segments are introduced into the main chain of PLA; blending modification, which involves blending PLA with other ductile polymer materials; and plasticizing modification, wherein small molecules or low molecular weight plasticizers are mixed with PLA to improve its toughness. Secondly, considering the large specific surface area and high reactivity of nanoparticles, incorporating nanoparticles enables rapid establishment of reactions with PLA molecules to form new groups and enhance their mechanical properties. In addition, when selecting materials to enhance the performance of PLA porous film, it is necessary to consider factors such as biodegradability, environmental friendliness, economic feasibility, and biocompatibility in order to improve the mechanical properties of the film while maintaining its excellent degradability and biocompatibility.

Furthermore, the utilization of a solvent in the fabrication process of porous membranes poses potential risks to human health. Therefore, it is imperative to prioritize the development of an environmentally-friendly alternative solvent. There are two aspects to consider: 1. Consider the utilization of low-toxic or non-toxic solvents, such as triethyl phosphate (TEP) and ethyl lactate; however, it is essential to take into account the solvent’s ability to dissolve PLA. 2. Ionic liquids can also be regarded as a viable alternative for substituting toxic solvents due to their advantageous characteristics of low volatility, environmental friendliness, and excellent structural tunability [159,160,161].

Thirdly, the incorporation of SC crystals can ameliorate the issue of slow crystallization and enhance the degree of crystallinity, thereby augmenting the thermal stability of PLA films. However, there is currently limited research in this area, necessitating further discussions on the impact of different preparation methods on sc crystals as well as their pore structure size and distribution.

Finally, the structure and size distribution of pores are particularly important for the application of porous membranes. Despite being influenced by various factors, achieving nanoscale pore structures remains a formidable challenge. Therefore, it is imperative to optimize the preparation process and enhance material structure for the fabrication of nanostructured porous membranes, thereby broadening their range of applications. The current literature predominantly employs a single processing method for the preparation of PLA porous membranes, which severely restricts the diversity of membrane structures and limits their potential applications. It is imperative to integrate multiple processing techniques in order to explore the fabrication of membrane materials with enhanced structure and performance. Furthermore, in order to better fulfill the demands of large-scale production, it is imperative to optimize processing equipment, enhance production efficiency, and expand the application scope of PLA porous film. As more researchers delve into PLA porous membranes, we are confident that these issues will be resolved and the potential applications for PLA porous membranes will continue to broaden.

## Figures and Tables

**Table 1:** Advantages and limitations of PLA porous membranes.

PLA Porous Membranes	Properties
advantage [10,11,12,13]	Low relative density
	High specific surface area
	Excellent thermal insulation
	Good permeability
	Biodegradable and biocompatibility
Limitation [14,15]	Low tensile strength
	High brittleness
	Poor heat resistance

**Table 2:** Electrospinning for PLA-based polymer film formation.

Polymer	Solvent	Voltage (kV)	Distance (cm)	Pore Size (μm)	Ref.
PVA/PLA	Water CHL/DMF	16	15	0.69 ± 0.09	[42]
PLA	Ac/DMF	25	25	0.315 ± 0.246	[46]
PLA/PCL	MC/DMF	12	15	/	[10]
PLA- KER	HFIP and Ac/CHL	12	15	1.02 ± 0.16	[47]
PLA/PCL	/	15	15	/	[48]
PLA/AgNWs	DCM	18	15	0.25–0.66	[49]
PLA	CHL	20	12	0.034–0.143	[50]
PLA/PANI	CHL	25	20	/	[51]
PLA/HA	HFIP	12	20	1.41–1.44	[52]
PLA/PVA	CHL/DCM	17.7	/	0.1–0.35	[21]
PLA/TPU	CHL/DMF	16	16	0.6417 ± 0.128	[53]
PLA	CHL/Ac	15	15	<3	[54]
PLA/Starch	CHL	6	15	2	[55]
PLA	DCM	25	15	0.1~0.16	[56]

KER, keratin; PCL, poly (e-caprolactone); AgNWs, silver nanowires; PANI, HA, Hyaluronic acid; polyaniline; TPU, thermoplastic polyurethanes; CHL, chloroform; DMF, *N*,*N*-dimethylformamide; AC, acetone; DCM, dichloromethane; DMAc, dimethylacetamide and dimethylformamide; MC, methylene chloride; HFIP, 1,1,1,3,3,3-Hexafluoro-2-propanol. Distance stands for tip-collector distance; “/” indicates that the relevant information is not mentioned in the literature.

**Table 3:** BF for PLA-based polymer film formation.

Polymer	Solvent	Ambient Humidity (%)	Pore Size (μm)	Degree of Order	Ref.
PLA	DCM	80%–85%	11.05–29.11	Normal	[65]
PM-*b*-PLA	DCM	95%	4.8	Normal	[58]
PLA-*b*-PS	DCM	/	0.9~1.2	High	[59]
H-PLA/GST	DCM	95%	3.2 ± 2.5	Normal	[12]
PLA/CS/BSA	CHL	/	<10	Normal	[67]
PtBA_90_-*b*-PFNEMA/PLA	CHL	80%	2.1	High	[68]
PLA/FAS-SiO_2_	DCM	56%	1~1.8	Low	[60]
PLLA/CQD	CHL	60%	1.85~30.59	Normal	[66]
PLA	CHL	99.9%	3~4	Normal	[69]
PLA	CHL	75%	2.0~2.8	High	[70]
PEG-PLA	CHL	70–80%	0.87	Normal	[71]
PLGA	DCM	70%	1.4~2.5	Normal	[72]

Solvent selection: the preparation of porous film by the BF necessitates the rapid volatilization of solvents. Therefore, it is essential to select organic solvents with high vapor pressure, low boiling points, and insolubility in water, such as dichloromethane and chloromethane; degree of order characterizes the ordered structure of pores; “/” indicates that the relevant information is not mentioned in the literature. GST, green synthesis of rutile titanium dioxide; CS, chitosan; BSA, bovine serum albumin; PtBA_90_-*b*-PFNEMA, poly (tert-butyl acrylate)-*b*-poly (2-[(Perfluorononenyl)oxy] ethyl methacrylate); FAS-SiO_2_, fluorinated silica nanoparticles; PEG, poly (ethylene glycol).

**Table 4:** Preparation parameters of PLA membranes fabricated via TIPS method.

Polymer	Diluent	Extractant	Pore Size (μm)	Porosity (%)	Ref.
PLLA	1,4-dioxane	Water	3~10	/	[88]
PLA	1,4-dioxane/*DMAC*	ethanol	0.3~0.5	87.6 ± 0.4	[90]
PLA	THF/water	ethanol	7.2~20.3	81.4~88.8	[91]
PLA	THF/MeOH	ethanol	0.17~1.44	81.4~90.6	[92]
PLA	1,4-dioxane	ethanol	26 ± 7	87.3	[93]
PLA	1,4-dioxane/water	/	/	>70	[94]
PLLA	1,4-dioxane/2-butanone/water	/	1.1~28.6	90–93%	[95]
PLA/CaSi/DCPD	CHL	ethanol	/	92.4~93.8	[96]
PCL/PLA/TCH	1,4-dioxane	/		78.57~81.53	[97]
PLLA	1,4-dioxane/DMAC	ethanol	/	46.9~83%	[89]
PLLA/PHEA-PLA	dioxane and deionised water	water	1–2	/	[98]
PLLA/PLA	dioxane/water	ethylalcohol	/	87~92%	[99]
PLA/nanoclay	1,4-dioxane	/	/	80%	[100]

DCPD, dicalcium phosphate dihydrate; PCL, poly (e-caprolactone); TCH, tetracycline hydrochloride; PHEA-PLA, α,β-poly[(*n*-hydroxyethyl)- DL-aspartamide] and PLA; THF, tetrahydrofuran; MeOH, methanol; “/” indicates that the relevant information is not mentioned in the literature.

**Table 6:** Comparison of the various PLA porous membranes prepared by different methods.

Method	The Formation Mechanism of Pores	Advantage	Disadvantage
Electrospinning [10,42,46,47]	Competition between electrostatic interactions and surface tension	The process is easy to control	Wide pore size distribution
Water droplet template method [12,59,65,119]	Low-boiling-point solvents evaporate, water droplets condense	The ordered porous structure can be formed at room temperature	Many influencing factors, difficult to control
TIPS [90,91,92,93]	The sites occupied by the diluent become micropores after their removal	Controlled pore structure and shape	Choosing the diluent is difficult, energy consumption is relatively high
NIPS [11,13,110,114]	Solvent–non-solvent exchange	Room temperature, diverse pore structure	The shape of the hole is not easy to control

**Table 7:** Applications of different PLA porous membranes in tissue engineering.

Polymer	Membrane Preparation Method	Hydrophilicity (°)	Application	Ref.
PLA/Kef	Electrospinning	/	Skin tissue engineering	[121]
PLA/ZrO_2_	Air-jet spinning technique (AJS)	/	Bone tissue engineering	[17]
PLA/Chitosan/gelatin	Centrifugally spinning	99–110	Soft tissue engineering	[128]
PLA/Iron	Fused filament fabrication	70.9–96.5	Bone tissue engineering	[129]
PANI/PLA	Electrospinning	36.7–61.6	Tissue engineering	[130]
PLA/SF	Electrospinning	112.81–142.49	Meniscus tissue engineering	[126]
PLA/HA/GO	Electrospinning	/	Tissue engineering	[131]
PLA/Gelatin	Electrospinning	135 ± 4	Polyester tissue engineering	[132]
PLA-PCL/rGO	Electrospinning	/	Tissue engineering	[133]
PLA/LAP/PEO	Electrospinning	26–111	Bone tissue engineering	[134]

Kef, Kefiran; PANI, polyaniline; SF, Silk Fibroin; HA, hydroxyapatite; GO, graphene oxide; rGO, reduced graphene oxide; LAP, laponite; PEO, polyethylene oxide; “/” indicates that the relevant information is not mentioned in the literature.

**Table 8:** Summary of oil–water separation for PLA porous membranes.

Absorbents	Membrane Preparation Method	Water Contact Angle (°)	Absorption Capacity (g/g)	Ref.
AKD-dipped PLA/Gly	Electrospinning	163	10	[142]
PLA/rGO	Electrospinning	139.2	/	[143]
PDLA/PLLA	Non-solvent-induced phase separation	168	9.7	[144]
PLA/PDA	/	151.7	36	[145]
PLA	Secondary phase separation	158	21	[146]
PLLA/PDLA	Non-solvent-induced phase separation	130.8	11	[147]
PLA	Thermally and non-solvent-induced phase separation	151	31.5	[148]
PLA@silicone semi-IPN	Electrospinning	158.7	83.9	[149]
PLA/CNT@LDH	Electrospinning	114.01	35	[150]
PLA/WO_3_/N-CQDs	Electrospinning	132.37	35.752	[151]
PLA/TiO_2_/MTS	Electrospinning	157.4 ± 0.9	/	[140]
PLA/PDA/AgNPs	Electrospinning	158.6 ± 1.2	/	[29]
PLA/CNTs	Electrospinning	157.3	114.01	[25]

AKD, alkyl ketene dimer; Gly, glycerol; PDA, polydopamine; IPN, interpenetrated network; CNT, carbon nanotubes; LDH, layered double hydroxides; WO_3_, tungsten oxide; N-CQDs, amino-functionalized carbon quantum dots; “/” indicates that the relevant information is not mentioned in the literature.

## Data Availability

Not applicable.

## References

[1] Wang C., Cheng S., Li H., Zuo D. (2021). Effect of different concentrations of spraying chitosan solution on structure and properties of PVDF porous membrane. Colloid. Polym. Sci..

[2] Wei N., Li Z., Li Q., Yang E., Xu R., Song X., Sun J., Dou C., Tian J., Cui H. (2021). Scalable and low-cost fabrication of hydrophobic PVDF/WS2 porous membrane for highly efficient solar steam generation. J. Colloid Interface Sci..

[3] Wang H., Guo X., Pei C., Dong W., Yao Y. (2022). Hydrophilic modification of polypropylene membrane via tannic and titanium complexation for high-efficiency oil/water emulsion separation driven by self-gravity. Polym. Eng. Sci..

[4] Zhu X., Zhu L., Xue J., Xue Q. (2023). Preparation of micro-nano particles modified discarded face-mask by a versatile thermocompression modification approach and its application to emulsion separation. Sep. Sci. Technol..

[5] Dong C., Xu X., Zhang J., Wang H., Xiang Y., Zhu H., Forsyth M., Lu S. (2022). Proton transport of porous triazole-grafted polysulfone membranes for high temperature polymer electrolyte membrane fuel cell. Int. J. Hydrogen Energy.

[6] Zhang Y., Liu F., Zhang S., Zhang Y., Liu S. (2012). Preparation of Nonstoichiometric Silica with Multi-Active Groups and Effect of Its Doping on Polysulfone Membrane Capabilities. Sep. Sci. Technol..

[7] Zhang S.-C., Liu T., Wang Y.-J. (2012). Porous and single-skinned polyethersulfone membranes support the growth of HepG2 cells: A potential biomaterial for bioartificial liver systems. J. Biomater. Appl..

[8] Zhou Q., Yang Y., Wang X., Wang Q., Wang S., Gao X., Gao C. (2020). Harvesting Microalgae Biomass Using Sulfonated Polyethersulfone (SPES)/PES Porous Membranes in Forward Osmosis Processes. J. Ocean Univ. China.

[9] Sakdaronnarong C., Srimarut N., Lucknakhul N., Na-songkla N., Jonglertjunya W. (2014). Two-step acid and alkaline ethanolysis/alkaline peroxide fractionation of sugarcane bagasse and rice straw for production of polylactic acid precursor. Biochem. Eng. J..

[10] Fan Y., Miao X., Hou C., Wang J., Lin J., Bian F. (2023). High tensile performance of PLA fiber-reinforced PCL composite via a synergistic process of strain and crystallization. Polymers.

[11] Gao A.L., Zhao Y.Q., Yang Q., Fu Y.Y., Xue L.X. (2016). Facile preparation of patterned petal-like PLA surfaces with tunable water micro-droplet adhesion properties based on stereo-complex co-crystallization from non-solvent induced phase separation processes. J. Mater. Chem..

[12] Shebi A., Lisa S. (2019). Evaluation of biocompatibility and bactericidal activity of hierarchically porous PLA-TiO_2_ nanocomposite films fabricated by breath-figure method. Mater. Chem. Phys..

[13] Su Y.Z., Zhao Y.Q., Zheng W.G., Yu H.W., Liu Y.F., Xu L.Q. (2020). Asymmetric Sc-PLA Membrane with Multi-scale Microstructures: Wettability, Antifouling, and Oil-Water Separation. ACS Appl. Mater. Interfaces.

[14] Hou Y., Jia H., Pan Y., Liu C., Shen C., Liu X. (2023). Porous poly (l-lactide)/poly (d-lactide) blend film with enhanced flexibility and heat resistance via constructing a regularly oriented pore structure. Macromolecules.

[15] Thiangtham S., Saito N., Manuspiya H. (2022). Asymmetric Porous and Highly Hydrophilic Sulfonated Cellulose/Biomembrane Functioning as a Separator in a Lithium-Ion Battery. ACS Appl. Energy Mater..

[16] Bakhshi R., Mohammadi-Zerankeshi M., Mehrabi-Dehdezi M., Alizadeh R., Labbaf S., Abachi P. (2023). Additive manufacturing of PLA-Mg composite scaffolds for hard tissue engineering applications. J. Mech. Behav. Biomed. Mater..

[17] Osorio-Arciniega R., Garcia-Hipolito M., Alvarez-Fregoso O., Alvarez-Perez M.A. (2021). Composite Fiber Spun Mat Synthesis and In Vitro Biocompatibility for Guide Tissue Engineering. Molecules.

[18] Heydari P., Parham S., Kharazi A.Z., Javanmard S.H., Asgary S. (2022). In Vitro Comparison Study of Plasma Treated Bilayer PGS/PCL and PGS/PLA Scaffolds for Vascular Tissue Engineering. Fibers Polym..

[19] Mokhtari N., Kharazi A.Z. (2021). Blood compatibility and cell response improvement of poly glycerol sebacate/poly lactic acid scaffold for vascular graft applications. J. Biomed. Mater. Res. Part A.

[20] Zhang F., Bambharoliya T., Xie Y., Liu L., Celik H., Wang L., Akkus O., King M.W. (2021). A hybrid vascular graft harnessing the superior mechanical properties of synthetic fibers and the biological performance of collagen filaments. Mater. Sci. Eng. C.

[21] Banitaba S.N., Gharehaghaji A.A., Jeddi A.A.A. (2021). Fabrication and characterization of hollow electrospun PLA structure through a modified electrospinning method applicable as vascular graft. Bull. Mater. Sci..

[22] Ebrahimifar M., Taherimehr M. (2021). Evaluation of in-vitro drug release of polyvinylcyclohexane carbonate as a CO2-derived degradable polymer blended with PLA and PCL as drug carriers. J. Drug Delivery Sci. Technol..

[23] Jeong H., Lim H., Lee D.Y., Song Y.-S., Kim B.-Y. (2021). Preparation and drug release behavior of nifedipine-loaded poly(lactic acid)/polyethylene glycol microcapsules. J. Nanosci. Nanotechnol..

[24] Seo K.H., Lee K.E., Yanilmaz M., Kim J. (2022). Exploring the diverse morphology of porous poly(Lactic Acid) fibers for developing long-term controlled antibiotic delivery systems. Pharmaceutic.

[25] Mo J., Wang Y., Lin J., Ke Y., Zhou C., Wang J., Wen J., Gan F., Wang L., Ma C. (2023). Polylactic acid/multi-wall carbon nanotubes composite fibrous membrane and their applications in oil-water separation. Surf. Interfaces.

[26] Zhang D., Jin X.-Z., Huang T., Zhang N., Qi X., Yang J.-h., Zhou Z., Wang Y. (2019). Electrospun fibrous membranes with dual-scaled porous structure: Super hydrophobicity, super lipophilicity, excellent water adhesion, and anti-icing for highly efficient oil adsorption/separation. ACS Appl. Mater. Interfaces.

[27] Barbosa J., Reizabal A., Correia D., Fidalgo-Marijuan A., Gonçalves R., Silva M., Lanceros-Mendez S., Costa C. (2020). Lithium-ion battery separator membranes based on poly (L-lactic acid) biopolymer. Mater. Today Energy.

[28] Zhong L., Gong X. (2019). Phase separation-induced superhydrophobic polylactic acid films. Soft Matter.

[29] Liu L., Yuan W. (2018). A hierarchical functionalized biodegradable PLA electrospun nanofibrous membrane with superhydrophobicity and antibacterial properties for oil/water separation. J. Chem..

[30] Hu S., Wu J., Cui Z., Si J., Wang Q., Peng X. (2020). Study on the mechanical and thermal properties of polylactic acid/hydroxyapatite@ polydopamine composite nanofibers for tissue engineering. J. Appl. Polym. Sci..

[31] Zhou J.-f., Wang Y.-g., Cheng L., Wu Z., Sun X.-d., Peng J. (2016). Preparation of polypyrrole-embedded electrospun poly (lactic acid) nanofibrous scaffolds for nerve tissue engineering. Neural Regener. Res..

[32] Mao Z., Li J., Huang W., Jiang H., Zimba B.L., Chen L., Wan J., Wu Q. (2018). Preparation of poly (lactic acid)/graphene oxide nanofiber membranes with different structures by electrospinning for drug delivery. RSC Adv..

[33] Buscemi S., Palumbo V.D., Maffongelli A., Fazzotta S., Palumbo F.S., Licciardi M., Fiorica C., Puleio R., Cassata G., Fiorello L. (2017). Electrospun PHEA-PLA/PCL Scaffold for Vascular Regeneration: A Preliminary in Vivo Evaluation. Trans. Proc..

[34] Afifi A.M., Nakajima H., Yamane H., Kimura Y., Nakano S. (2009). Fabrication of Aligned Poly (l-lactide) Fibers by Electrospinning and Drawing. Macromol. Mater. Eng..

[35] Samatham R., Kim K. (2006). Electric current as a control variable in the electrospinning process. Polym. Eng. Sci..

[36] Moon S., Choi J., Farris R.J. (2008). Highly porous polyacrylonitrile/polystyrene nanofibers by electrospinning. Fibers Polym..

[37] Park J.Y., Lee I.H. (2011). Controlled release of ketoprofen from electrospun porous polylactic acid (PLA) nanofibers. J. Polym. Res..

[38] Zhang H., Niu Q., Wang N., Nie J., Ma G. (2015). Thermo-sensitive drug controlled release PLA core/PNIPAM shell fibers fabricated using a combination of electrospinning and UV photo-polymerization. Eur. Polym. J..

[39] Tian R., Zhang P., Lv R., Na B., Liu Q., Ju Y. (2015). Formation of highly porous structure in the electrospun polylactide fibers by swelling-crystallization in poor solvents. RSC Adv..

[40] Bognitzki M., Frese T., Steinhart M., Greiner A., Wendorff J.H., Schaper A., Hellwig M. (2001). Preparation of fibers with nanoscaled morphologies: Electrospinning of polymer blends. Polym. Eng. Sci..

[41] Yang H., Wang L., Xiang C., Li L. (2018). Electrospun porous PLLA and poly (LLA-co-CL) fibers by phase separation. New J. Chem..

[42] Zhong G., Qiu M., Zhang J., Jiang F., Yue X., Huang C., Zhao S., Zeng R., Zhang C., Qu Y. (2023). Fabrication and characterization of PVA@PLA electrospinning nanofibers embedded with Bletilla striata polysaccharide and Rosmarinic acid to promote wound healing. Int. J. Biol. Macromol..

[43] Chen X., Li H.H., Lu W.P., Guo Y.C. (2021). Antibacterial Porous Coaxial Drug-Carrying Nanofibers for Sustained Drug-Releasing Applications. Nanomaterials.

[44] Tian L., Prabhakaran M.P., Hu J., Chen M., Besenbacher F., Ramakrishna S. (2015). Coaxial electrospun poly(lactic acid)/silk fibroin nanofibers incorporated with nerve growth factor support the differentiation of neuronal stem cells. RSC Adv..

[45] Zhang P., Tian R., Lv R., Na B., Liu Q. (2015). Water-permeable polylactide blend membranes for hydrophilicity-based separation. Chem. Eng. J..

[46] Lo J.S.C., Daoud W., Tso C.Y., Lee H.H., Firdous I., Deka B.J., Lin C.S.K. (2022). Optimization of polylactic acid-based medical textiles via electrospinning for healthcare apparel and personal protective equipment. Sustain. Chem. Pharm..

[47] Ertek D.A., Sanli N.O., Menceloglu Y.Z., Avaz Seven S. (2023). Environmentally friendly, antibacterial materials from recycled keratin incorporated electrospun PLA films with tunable properties. Eur. Polym. J..

[48] Scaffaro R., Lopresti F., Botta L. (2017). Preparation, characterization and hydrolytic degradation of PLA/PCL co-mingled nanofibrous mats prepared via dual-jet electrospinning. Eur. Polym. J..

[49] Zhao Y., Lu Q., Wu J., Zhang Y., Guo J., Yu J., Shu X., Chen Q. (2023). Flexible, robust and self-peeling PLA/AgNWs nanofiber membranes with photothermally antibacterial properties for wound dressing. Appl. Surf. Sci..

[50] Gulzar S., Tagrida M., Nilsuwan K., Prodpran T., Benjakul S. (2022). Electrospinning of gelatin/chitosan nanofibers incorporated with tannic acid and chitooligosaccharides on polylactic acid film: Characteristics and bioactivities. Food Hydrocolloids.

[51] Guo Y., Ghobeira R., Sun Z., Shali P., Morent R., De Geyter N. (2022). Atmospheric pressure plasma jet treatment of PLA/PAni solutions: Enhanced morphology, improved yield of electrospun nanofibers and concomitant doping behaviour. Polymers.

[52] Niu Y., Stadler F.J., Fang J., Galluzzi M. (2021). Hyaluronic acid-functionalized poly-lactic acid (PLA) microfibers regulate vascular endothelial cell proliferation and phenotypic shape expression. Colloids Surf. B.

[53] Wu Z.C., Zhang Z.J., Wei W., Yin Y.Q., Huang C.X., Ding J., Duan Q.S. (2022). Investigation of a novel poly (lactic acid) porous material toughened by thermoplastic polyurethane. J. Mater. Sci..

[54] Scaffaro R., Maio A., Gulino E.F., Micale G.D.M. (2020). PLA-based functionally graded laminates for tunable controlled release of carvacrol obtained by combining electrospinning with solvent casting. React. Funct. Polym..

[55] Gutiérrez-Sánchez M., Escobar-Barrios V.A., Pozos-Guillén A., Escobar-García D.M. (2019). RGD-functionalization of PLA/starch scaffolds obtained by electrospinning and evaluated in vitro for potential bone regeneration. Mater. Sci. Eng. C.

[56] Min T., Zhou L., Sun X., Du H., Bian X., Zhu Z., Wen Y. (2022). Enzyme-responsive food packaging system based on pectin-coated poly (lactic acid) nanofiber films for controlled release of thymol. Food Res. Int..

[57] François B., Pitois O., François J. (1995). Polymer films with a self-organized honeycomb morphology. Adv. Mater..

[58] Li Q.Z., Zhang G.Y., Huang J., Zhao Q.L., Wei L.H., He Z.H., Ma Z. (2011). Synthesis and Property of Well-defined Polymethylene/Polylactide Diblock Copolymer. Acta Chim. Sinica.

[59] Bertrand A., Bousquet A., Lartigau-Dagron C., Billon L. (2016). Hierarchically porous bio-inspired films prepared by combining “breath figure” templating and selectively degradable block copolymer directed self-assembly. Chem. Commun..

[60] Chen Y., Gao X.-R., Huang H.-D., Xu L., Ji X., Zhong G.-J., Lin H., Li Z.-M. (2021). Superhydrophobic, Self-Cleaning, and Robust Properties of Oriented Polylactide Imparted by Surface Structuring. ACS Sustain. Chem. Eng..

[61] Bai H., Du C., Zhang A.J., Li L. (2013). Breath Figure Arrays: Unconventional Fabrications, Functionalizations, and Applications. Angew. Chem. Int. Ed..

[62] Escale P., Rubatat L., Billon L., Save M. (2012). Recent advances in honeycomb-structured porous polymer films prepared via breath figures. Eur. Polym. J..

[63] Huang J., Zhu J., Sun W., Ji J. (2020). Versatile and functional surface patterning of in situ breath figure pore formation via solvent treatment. ACS Appl. Mater. Interfaces.

[64] Kong Q., Li Z., Ding F., Ren X. (2021). Hydrophobic N-halamine based POSS block copolymer porous films with antibacterial and resistance of bacterial adsorption performances. Chem. Eng. J..

[65] Preuksarattanawut C., Nisaratanaporn E., Siralertmukul K. (2019). Highly ordered porous PLA films prepared by breath figure method. J. Met. Mater. Miner..

[66] Guo Y., Sun X., Wang R., Tang H., Wang L., Zhang L., Qin S. (2022). Construction of porous poly (l-lactic acid) surface via carbon quantum dots-assisted static Breath-Figures method. Colloids Surf. A.

[67] Ding L.Y., Ju Y.L., Sun W., Chen J. (2018). Preparation of Functional Patterned Protein Arrays via the Combination of Inverse Emulsion and Breath Figure Method. Chem. J. Chin. Univ. Chin..

[68] Qin S., Li H., Yuan W.Z., Zhang Y.M. (2012). Fabrication of polymeric honeycomb microporous films: Breath figures strategy and stabilization of water droplets by fluorinated diblock copolymer micelles. J. Mater. Sci..

[69] Cheng K.-Y., Chang C.-H., Yang Y.-W., Liao G.-C., Liu C.-T., Wu J.-S. (2017). Enhancement of cell growth on honeycomb-structured polylactide surface using atmospheric-pressure plasma jet modification. Appl. Surf. Sci..

[70] Yin H., Zhan F., Li Z., Huang H., Marcasuzaa P., Luo X., Feng Y., Billon L. (2021). CO_2_-Triggered ON/OFF Wettability Switching on Bioinspired Polylactic Acid Porous Films for Controllable Bioadhesion. Biomac..

[71] Yao B., Zhu Q., Yao L., Hao J. (2015). Fabrication of honeycomb-structured poly(ethylene glycol)-block-poly(lactic acid) porous films and biomedical applications for cell growth. Appl. Surf. Sci..

[72] Ponnusamy T., Lawson L.B., Freytag L.C., Blake D.A., Ayyala R.S., John V. (2012). In vitro degradation and release characteristics of spin coated thin films of PLGA with a “breath figure” morphology. Biomatter.

[73] Loeb S., Sourirajan S. (1962). High-flow semipermeable membranes for separation of water from saline solutions. Adv. Chem. Ser..

[74] Fijol N., Abdelhamid H.N., Pillai B., Hall S.A., Thomas N., Mathew A.P. (2021). 3D-printed monolithic biofilters based on a polylactic acid (PLA)—Hydroxyapatite (HAp) composite for heavy metal removal from an aqueous medium. RSC Adv..

[75] Xing Q., Dong X., Li R., Yang H., Han C.C., Wang D. (2013). Morphology and performance control of PLLA-based porous membranes by phase separation. Polymer.

[76] Figoli A., Marino T., Galiano F. (2016). Polymeric membranes in biorefinery. Membr. Technol. Biorefining.

[77] Lloyd D.R., Kinzer K.E., Tseng H. (1990). Microporous membrane formation via thermally induced phase separation. I. Solid-liquid phase separation. J. Membr. Sci..

[78] Lloyd D.R., Kim S.S., Kinzer K. (1991). Microporous membrane formation via thermally-induced phase separation. II. Liquid—Liquid phase separation. J. Membr. Sci..

[79] Kim S.S., Lloyd D. (1991). Microporous membrane formation via thermally-induced phase separation. III. Effect of thermodynamic interactions on the structure of isotactic polypropylene membranes. J. Membr. Sci..

[80] Lim G.B., Kim S.S., Ye Q., Wang Y.F., Lloyd D. (1991). Microporous membrane formation via thermally-induced phase separation. IV. Effect of isotactic polypropylene crystallization kinetics on membrane structure. J. Membr. Sci..

[81] Kim S.S., Lim G.B., Alwattari A.A., Wang Y.F., Lloyd D. (1991). Microporous membrane formation via thermally-induced phase separation. V. Effect of diluent mobility and crystallization on the structure of isotactic polypropylene membranes. J. Membr. Sci..

[82] Alwattari A.A., Lloyd D. (1991). Microporous membrane formation via thermally-induced phase separation. VI. Effect of diluent morphology and relative crystallization kinetics on polypropylene membrane structure. J. Membr. Sci..

[83] Lloyd D.R., Lim G. (1993). Microporous membrane formation via thermally-induced phase separation. VII. Effect of dilution, cooling rate, and nucleating agent addition on morphology. J. Membr. Sci..

[84] Chinyerenwa A.C., Wang H., Zhang Q., Zhuang Y., Munna K.H., Ying C., Yang H., Xu W. (2018). Structure and thermal properties of porous polylactic acid membranes prepared via phase inversion induced by hot water droplets. Polymer.

[85] Rajabzadeh S., Maruyama T., Sotani T., Matsuyama H.J.S., Technology P. (2008). Preparation of PVDF hollow fiber membrane from a ternary polymer/solvent/nonsolvent system via thermally induced phase separation (TIPS) method. Sep. Purif. Technol..

[86] Cui Z., Hassankiadeh N.T., Zhuang Y., Drioli E., Lee Y.M. (2015). Crystalline polymorphism in poly(vinylidenefluoride) membranes. Prog. Polym. Sci..

[87] Liu Q., Tian S., Zhao C., Chen X., Lei I., Wang Z., Ma P.X. (2015). Porous nanofibrous poly(l-lactic acid) scaffolds supporting cardiovascular progenitor cells for cardiac tissue engineering. Acta Biomater..

[88] Tanaka T., Lloyd D. (2004). Formation of poly (L-lactic acid) microfiltration membranes via thermally induced phase separation. J. Membr. Sci..

[89] Soltanolkottabi F. (2022). Application of Fourier’s law in thermally induced phase separation (TIPS) process for porous poly(L-lactide) films. Polym. Bull..

[90] Hsu S.h., Huang S., Wang Y.C., Kuo Y.C. (2013). Novel nanostructured biodegradable polymer matrices fabricated by phase separation techniques for tissue regeneration. Acta Biomater..

[91] Önder Ö.C., Yilgör E., Yilgör I. (2016). Fabrication of rigid poly(lactic acid) foams via thermally induced phase separation. Polymer.

[92] Onder O.C., Yilgor E., Yilgor I. (2019). Critical parameters controlling the properties of monolithic poly(lactic acid) foams prepared by thermally induced phase separation. J. Polym. Sci. Part B-Polym. Phys..

[93] Gay S., Lefebvre G., Bonnin M., Nottelet B., Boury F., Gibaud A., Calvignac B. (2018). PLA scaffolds production from Thermally Induced Phase Separation: Effect of process parameters and development of an environmentally improved route assisted by supercritical carbon dioxide. J. Supercrit. Fluids.

[94] Chen J.-S., Tu S.-L., Tsay R.-Y. (2010). A morphological study of porous polylactide scaffolds prepared by thermally induced phase separation. J. Taiwan Inst. Chem. Eng..

[95] Kanno T., Uyama H. (2017). Unique leafy morphology of poly(lactic acid) monoliths controlled via novel phase separation technology. RSC Adv..

[96] Gandolfi M.G., Zamparini F., Degli Esposti M., Chiellini F., Aparicio C., Fava F., Fabbri P., Taddei P., Prati C. (2018). Polylactic acid-based porous scaffolds doped with calcium silicate and dicalcium phosphate dihydrate designed for biomedical application. Mater. Sci. Eng..

[97] Farzamfar S., Naseri-Nosar M., Sahrapeyma H., Ehterami A., Goodarzi A., Rahmati M., Lakalayeh G.A., Ghorbani S., Vaez A., Salehi M. (2019). Tetracycline hydrochloride-containing poly (epsilon-caprolactone)/poly lactic acid scaffold for bone tissue engineering application: In vitro and in vivo study. Int. J. Polym. Mater. Polym. Biomater..

[98] Carfì Pavia F., Palumbo F.S., La Carrubba V., Bongiovì F., Brucato V., Pitarresi G., Giammona G. (2016). Modulation of physical and biological properties of a composite PLLA and polyaspartamide derivative obtained via thermally induced phase separation (TIPS) technique. Mater. Sci. Eng. C.

[99] La Carrubba V., Pavia F.C., Brucato V., Piccarolo S. (2008). PLLA/PLA scaffolds prepared via Thermally Induced Phase Separation (TIPS): Tuning of properties and biodegradability. Int. J. Mater. Form..

[100] Salehi M., Bastami F., Rad M.R., Nokhbatolfoghahaei H., Paknejad Z., Nazeman P., Hassani A., Khojasteh A. (2021). Investigation of cell-free poly lactic acid/nanoclay scaffolds prepared via thermally induced phase separation technique containing hydroxyapatite nanocarriers of erythropoietin for bone tissue engineering applications. Polym. Adv. Technol..

[101] Dong X., Lu D., Harris T.A., Escobar I. (2021). Polymers and solvents used in membrane fabrication: A review focusing on sustainable membrane development. Membranes.

[102] Guillen G.R., Pan Y.J., Li M.H., Hoek E.M.V. (2011). Preparation and Characterization of Membranes Formed by Nonsolvent Induced Phase Separation: A Review. Ind. Eng. Chem. Res..

[103] Hu R., Pi Y., Wang N., Zhang Q., Feng J., Xu W., Dong X., Wang D., Yang H. (2016). The formation of the S-shaped edge-on lamellae on the thin porous polylactic acid membrane via phase separation induced by water microdroplets. J. Appl. Polym. Sci..

[104] Moriya A., Maruyama T., Ohmukai Y., Sotani T., Matsuyama H. (2009). Preparation of poly (lactic acid) hollow fiber membranes via phase separation methods. J. Membr. Sci..

[105] Al Tawil E., Monnier A., Nguyen Q.T., Deschrevel B. (2018). Microarchitecture of poly(lactic acid) membranes with an interconnected network of macropores and micropores influences cell behavior. Eur. Polym. J..

[106] Kang Y., Chen P., Shi X.T., Zhang G.C., Wang C.L. (2018). Preparation of open-porous stereocomplex PLA/PBAT scaffolds and correlation between their morphology, mechanical behavior, and cell compatibility. RSC Adv..

[107] Fotsing E.R., Rezabeigi E., Ross A., Wood-Adams P.M., Drew R. (2019). Acoustical characteristics of ultralight polylactic acid foams fabricated via solution phase inversion. J. Porous Mater..

[108] Liu W., Huang N., Yang J., Peng L., Li J., Chen W. (2022). Characterization and application of porous polylactic acid films prepared by nonsolvent-induced phase separation method. Food Chem..

[109] Li G., Wang L., Lei X., Peng Z., Wan T., Maganti S., Huang M., Murugadoss V., Seok I., Jiang Q. (2022). Flexible, yet robust polyaniline coated foamed polylactic acid composite electrodes for high-performance supercapacitors. Adv. Compos. Hybrid Mater..

[110] Gao A., Zhang G., Zhao S., Cui J., Yan Y. (2019). A solution for trade-off phenomenon based on symmetric-like membrane with nano-scale pore structure. Sep. Purif. Technol..

[111] Ampawan S., Phreecha N., Chantarak S., Chinpa W. (2023). Selective separation of dyes by green composite membrane based on polylactide with carboxylated cellulose microfiber from empty fruit bunch. Int. J. Biol. Macromol..

[112] Zhu L.J., Liu F., Yu X.M., Xue L.X. (2015). Poly(Lactic Acid) Hemodialysis Membranes with Poly(Lactic Acid)-block-Poly(2-Hydroxyethyl Methacrylate) Copolymer As Additive: Preparation, Characterization, and Performance. ACS Appl. Mater. Interfaces.

[113] Liu F., Li B.B., Sun D., Li F.G., Pei X.Y. (2022). The effect of chitosan (CS) coagulation bath on structure and performance of polylactic acid (PLA) microfiltration membrane. Korean J. Chem. Eng..

[114] Shu Z., Zhang C.C., Yan L.Z., Lei H.Q., Peng C.X., Liu S., Fan L.H., Chu Y.Y. (2023). Antibacterial and osteoconductive polycaprolactone/polylactic acid/nano-hydroxyapatite/Cu@ZIF-8 GBR membrane with asymmetric porous structure. Int. J. Biol. Macromol..

[115] Hazra R.S., Dutta D., Mamnoon B., Nair G., Knight A., Mallik S., Ganai S., Reindl K., Jiang L., Quadir M. (2021). Polymeric Composite Matrix with High Biobased Content as Pharmaceutically Relevant Molecular Encapsulation and Release Platform. ACS Appl. Mater. Interfaces.

[116] Thiangtham S., Runt J., Saito N., Manuspiya H. (2020). Fabrication of biocomposite membrane with microcrystalline cellulose (MCC) extracted from sugarcane bagasse by phase inversion method. Cellulose.

[117] Moriya A., Shen P., Ohmukai Y., Maruyama T., Matsuyama H. (2012). Reduction of fouling on poly(lactic acid) hollow fiber membranes by blending with poly(lactic acid)–polyethylene glycol–poly(lactic acid) triblock copolymers. J. Membr. Sci..

[118] Han W., Ren J., Xuan H., Ge L. (2018). Controllable degradation rates, antibacterial, free-standing and highly transparent films based on polylactic acid and chitosan. Colloids Surf. A.

[119] Song X., Yin X., Cai Y., Wei Q., Liu W. (2018). Preparation and characterization of PLA porous film by breath figure method. New Chem. Mater..

[120] Hua M., Chen D., Xu Z., Fang Y., Song Y. (2022). Fabrication of high-expansion, fully degradable polylactic acid-based foam with exponent oil/water separation. J. Appl. Polym. Sci..

[121] Lopresti F., Campora S., Tirri G., Capuana E., Carfì Pavia F., Brucato V., Ghersi G., La Carrubba V. (2021). Core-shell PLA/Kef hybrid scaffolds for skin tissue engineering applications prepared by direct kefiran coating on PLA electrospun fibers optimized via air-plasma treatment. Mater. Sci. Eng. C.

[122] Farahani A., Zarei-Hanzaki A., Abedi H.R., Tayebi L., Mostafavi E. (2021). Polylactic Acid Piezo-Biopolymers: Chemistry, Structural Evolution, Fabrication Methods, and Tissue Engineering Applications. J. Funct. Biomater..

[123] Nie T., Xue L., Ge M., Ma H., Zhang J. (2016). Fabrication of poly(L-lactic acid) tissue engineering scaffolds with precisely controlled gradient structure. Mater. Lett..

[124] Pinto A.M., Moreira S., Gonçalves I.C., Gama F.M., Mendes A.M., Magalhães F.D. (2013). Biocompatibility of poly (lactic acid) with incorporated graphene-based materials. Colloids Surf. B.

[125] Li J., Liu F., Yu X., Wu Z., Wang Y., Xiong Z., He J. (2016). APTES assisted surface heparinization of polylactide porous membranes for improved hemocompatibility. RSC Adv..

[126] Promnil S., Ruksakulpiwat C., Numpaisal P., Ruksakulpiwat Y. (2022). Electrospun Poly(lactic acid) and Silk Fibroin Based Nanofibrous Scaffold for Meniscus Tissue Engineering. Polymers.

[127] Wang C., Wang H., Chen Q., Gang H., Zhou Y., Gu S., Liu X., Xu W., Zhang B., Yang H. (2022). Polylactic acid scaffold with directional porous structure for large-segment bone repair. Int. J. Biol. Macromol..

[128] Eftekhari-pournigjeh F., Saeed M., Rajabi S., Tamimi M., Pezeshki-Modaress M. (2023). Three-dimensional biomimetic reinforced chitosan/gelatin composite scaffolds containing PLA nano/microfibers for soft tissue engineering application. Int. J. Biol. Macromol..

[129] Jiang D., Ning F., Wang Y. (2021). Additive manufacturing of biodegradable iron-based particle reinforced polylactic acid composite scaffolds for tissue engineering. J. Mater. Process. Technol..

[130] Liu R.T., Zhang S.Y., Zhao C., Yang D., Cui T.T., Liu Y.D., Min Y.G. (2021). Regulated Surface Morphology of Polyaniline/Polylactic Acid Composite Nanofibers via Various Inorganic Acids Doping for Enhancing Biocompatibility in Tissue Engineering. Nanoscale Res. Lett..

[131] Oktay B., Ahlatcıoğlu Özerol E., Sahin A., Gunduz O., Ustundag C. (2022). Production and characterization of PLA/HA/GO nanocomposite scaffold. Chem. Sel..

[132] Bogdanova A., Pavlova E., Polyanskaya A., Volkova M., Biryukova E., Filkov G., Trofimenko A., Durymanov M., Klinov D., Bagrov D. (2023). Acceleration of Electrospun PLA Degradation by Addition of Gelatin. Int. J. Mol. Sci..

[133] Kovaleva P.A., Pariy I.O., Chernozem R.V., Zadorozhnyy M.Y., Permyakova E.S., Kolesnikov E.A., Surmeneva M.A., Surmenev R.A., Senatov F.S. (2022). Shape memory effect in hybrid polylactide-based polymer scaffolds functionalized with reduced graphene oxide for tissue engineering. Eur. Polym. J..

[134] Orafa Z., Irani S., Zamanian A., Bakhshi H., Nikukar H., Ghalandari B. (2021). Coating of Laponite on PLA Nanofibrous for Bone Tissue Engineering Application. Macromol. Res..

[135] Shan X., Huang P., Yang L., Feng R., Wang Z. (2022). Robust polypropylene/ethylene-propylene-diene terpolymer thermoplastic vulcanizates film for green oil-water separation. J. Polym. Res..

[136] Yin Z., Pan Y., Bao M., Li Y. (2021). Superhydrophobic magnetic cotton fabricated under low carbonization temperature for effective oil/water separation. Sep. Purif. Technol..

[137] Zeng X., Zhao L., Fan G., Yan C. (2021). Experimental study on the design of light phase outlets for a novel axial oil-water separator. Chem. Eng. Res. Des..

[138] Yin C., Meng Z. (2022). Evaluation of oil-water sepa tion performance of photocatalytic self-cleaning chitosan/aminated graphene/TiO_2_ superhydrophobic mesh membrane. Fresenius Environ.Bull..

[139] Mittag A., Rahman M.M., Hafez I., Tajvidi M. (2022). Development of Lignin-Containing Cellulose Nanofibrils Coated Paper-Based Filters for Effective Oil-Water Separation. Membranes.

[140] Zhou Z., Liu L., Yuan W. (2019). A superhydrophobic poly (lactic acid) electrospun nanofibrous membrane surface-functionalized with TiO_2_ nanoparticles and methyltrichlorosilane for oil/water separation and dye adsorption. New J. Chem..

[141] Sun X., Xue B., Tian Y., Qin S., Xie L. (2018). 3D porous poly (l-lactic acid) materials with controllable multi-scale microstructures and their potential application in oil-water separation. Appl. Surf. Sci..

[142] Eang C., Opaprakasit P. (2020). Electrospun Nanofibers with Superhydrophobicity Derived from Degradable Polylactide for Oil/Water Separation Applications. J. Polym. Environ..

[143] Du G., Duan Y., Yuan Q., Hu S. (2022). Preparation of PLA/rGO nanofiber membrane by electrospinning method and its application in oil-water separation. J. Funct. Mater..

[144] Guo Y., Sun X., Xue B., Zhou Y., Xie L., Zheng Q. (2023). Carbon quantum dots-driven surface morphology transformation towards superhydrophobic poly(lactic acid) film. Colloids Surf. A.

[145] Zeng Q., Ma P., Su X., Lai D., Lai X., Zeng X., Li H. (2020). Facile fabrication of superhydrophobic and magnetic poly (lactic acid) nonwoven fabric for oil–water separation. Ind. Eng. Chem..

[146] Wang Y., Yang H., Chen Z., Chen N., Pang X., Zhang L., Minari T., Liu X., Liu H., Chen J. (2018). Recyclable oil-absorption foams via secondary phase separation. ACS Sustain. Chem. Eng..

[147] Zhu C., Jiang W., Hu J., Sun P., Li A., Zhang Q. (2020). Polylactic acid nonwoven fabric surface modified with stereocomplex crystals for recyclable use in oil/water separation. ACS Appl. Polym. Mater..

[148] Wang X.L., Pan Y.M., Liu X.H., Liu H., Li N., Liu C.T., Schubert D.W., Shen C.Y. (2019). Facile Fabrication of Superhydrophobic and Eco-Friendly Poly(lactic acid) Foam for Oil-Water Separation via Skin Peeling. ACS Appl. Mater. Interfaces.

[149] Mo J., Wang Y., Wang J., Zhao J., Ke Y., Han S., Gan F., Wang L., Ma C. (2022). Hydrophobic/oleophilic polylactic acid electrospun fibrous membranes with the silicone semi-interpenetrated networks for oil-water separation. J. Mater. Sci..

[150] Liu W., Wu X., Liu S., Cheng X., Zhang C. (2022). CNT@LDH functionalized poly(lactic acid) membranes with super oil-water separation and real-time press sensing properties. Polym. Compos..

[151] Nugraha M.W., Wirzal M.D.H., Ali F., Roza L., Sambudi N.S. (2021). Electrospun polylactic acid/tungsten oxide/amino-functionalized carbon quantum dots (PLA/WO_3_/N-CQDs) fibers for oil/water separation and photocatalytic decolorization. J. Environ. Sci. Chem. Eng..

[152] Huang Y., Wang Y., Fu Y. (2022). 3D hierarchical biobased gel electrolyte with superior ionic conductivity and flame resistance for suppressing lithium dendrites via alloying and sieving mechanisms. Composites Part B.

[153] Khan N.M., Kufian M.Z., Samsudin A.S. (2023). The Correlation of Free Ions with the Conduction Phase of 1-Ethyl-3-methylimidazolium Chloride in Gel Polymer Electrolyte-Based PMMA/PLA Blend Doped with LiBOB. J. Electron. Mater..

[154] Wan Y., Yang S., Peng M., Gama M., Yang Z., Deng X., Zhou J., Ouyang C., Luo H. (2020). Controllable synthesis of biomimetic nano/submicro-fibrous tubes for potential small-diameter vascular grafts. J Mater Chem B.

[155] Cheng S., Hang C., Ding L., Jia L., Tang L., Mou L., Qi J., Dong R., Zheng W., Zhang Y. (2020). Electronic Blood Vessel. Matter.

[156] Domalik-Pyzik P., Morawska-Chochól A. (2022). Preliminary Results on Heparin-Modified Double-Layered PCL and PLA-Based Scaffolds for Tissue Engineering of Small Blood Vessels. J. Funct. Biomater..

[157] Khalifehzadeh R., Ciridon W., Ratner B.D. (2018). Surface fluorination of polylactide as a path to improve platelet associated hemocompatibility. Acta Biomater..

[158] Leyva-Verduzco A.A., Castillo-Ortega M.M., Chan-Chan L.H., Silva-Campa E., Galaz-Méndez R., Vera-Graziano R., Encinas-Encinas J.C., Del Castillo-Castro T., Rodríguez-Félix D.E., Santacruz-Ortega H. (2020). Electrospun tubes based on PLA, gelatin and genipin in different arrangements for blood vessel tissue engineering. Polym. Bull..

[159] Medina-Gonzalez Y., Aimar P., Lahitte J.-F., Remigy J. (2011). Towards green membranes: Preparation of cellulose acetate ultrafiltration membranes using methyl lactate as a biosolvent. Int. J. Sustain. Eng..

[160] Ratti R. (2014). Ionic liquids: Synthesis and applications in catalysis. Adv. Chem.

[161] Rogers R.D., Seddon K.R. (2003). Ionic liquids—Solvents of the future?. Science.

